# *Porphyromonas gingivalis* induces intestinal inflammation through gingipain-dependent gut microbiome dysbiosis

**DOI:** 10.1186/s40168-026-02389-7

**Published:** 2026-04-02

**Authors:** Ming Li, Jiyun Cui, Ru Qu, Rulong Liu, Yifan Sun, Ping Li, Juan Liu, Adrian Low, Xiaochang Huang, Fei Gan, Zhenjiang Zech Xu

**Affiliations:** 1 Shenzhen Hospital, Southern Medical University, Shenzhen, Guangdong China; 2 College of Food and Bioengineering, Bengbu University, Bengbu, China; 3 State Key Laboratory of Food Science and Resources, No. 235 Nanjing East Road, Nanchang, 330047 Jiangxi China; 4 Hubei Key Laboratory of Cell Homeostasis, College of Life Science, TaiKang Center for Life and Medical Sciences, Wuhan University, Wuhan, China; 5 Institute of Biological Resources, Jiangxi Academy of Sciences, Nanchang, 330096 China; 6 Department of Medicine, Yong Loo Lin School of Medicine, National University of Singapore, MD6 Centre for Translational Medicine, 14 Medical Drive, Singapore, 117599 Singapore

**Keywords:** *Porphyromonas gingivalis*, Intestinal inflammation, Th17 cells, Gingipain, Immunization

## Abstract

**Background:**

*Porphyromonas gingivalis* (Pg), a key pathogen in periodontitis, is implicated in various systemic diseases such as pancreatic cancer and Alzheimer’s disease. However, as a periodontal pathogen that can directly enter the lower gastrointestinal tract via saliva, its potential impact on the gut microbiome, intestinal inflammation, and its underlying mechanisms remains largely elusive.

**Results:**

Here, we observed that oral administration of Pg exacerbates intestinal inflammation in mice by inducing gut microbiome dysbiosis, increasing Th17 cells and the release of pro-inflammatory cytokines. Inhibition of Th17 activity with GSK805 or an anti-IL-17A blocking antibody mitigated this inflammatory response, highlighting the mediating role of Th17 cells. Gingipains, the virulence factors of Pg, played a crucial role in this process. Sequential knockout of gingipain genes revealed a gradual reduction in inflammatory phenotypes, with statistically significant alleviation observed when all three gingipain genes were deleted. Co-housing experiments showed that gut microbiota remodeling effectively protected against Th17-driven inflammatory response. Furthermore, immunization with inactivated Pg effectively prevented gut microbiome dysbiosis and Th17 cell-mediated inflammation.

**Conclusion:**

Our findings suggest that Pg may exacerbate intestinal inflammation, potentially via its gingipain virulence proteases, which are linked to gut microbiota dysbiosis and enhanced Th17-mediated immune responses. These results suggest that gingipains could be promising targets for further investigation in Pg-associated intestinal disorders.

Video Abstract

**Supplementary Information:**

The online version contains supplementary material available at https://doi.org/10.1186/s40168-026-02389-7.

## Introduction

Recent research suggests the potential existence of a “mouth-gut axis” in the pathogenesis of gastrointestinal diseases [1]. Multiple epidemiological studies have linked periodontitis, a chronic inflammatory disease initiated by dysbiotic oral microbiota, to the risk of inflammatory bowel disease (IBD) [2]. Concurring with epidemiological findings, other periodontal pathobionts have been reported to translocate to the gut [3, 4], where their ectopic colonization and immunomodulatory effects exacerbate colitis progression in murine models, highlighting their potential impact on gastrointestinal health [5, 6]. As a primary oral pathogen responsible for periodontal disease, Pg is implicated in various systemic diseases, including rheumatoid arthritis [7], insulin resistance [8], and increased cardiovascular disease risk [9]. Epidemiological studies have also highlighted a correlation between serum anti-Pg IgG antibodies and overall orodigestive disease mortality [10], while Pg has been found to be enriched in fecal samples from CRC patients [11]. Additionally, some studies have indicated that oral administration of Pg might alter the composition of the gut microbiota in healthy mice [12]. Nevertheless, the impact of Pg on intestinal inflammation and its underlying mechanisms remain to be further validated and explored.

Gingipain proteases, the major virulence factors of Pg, are cysteine proteases with arginine-specific (RgpA and RgpB) and lysine-specific (Kgp) activity. Beyond facilitating tissue invasion and immune evasion in the oral cavity, these enzymes have been detected in extraoral tissues, suggesting their involvement in systemic diseases. For example, gingipains have been identified in the brains of Alzheimer’s disease patients, where they are thought to contribute to amyloid-beta accumulation and neuroinflammation [13], and similarly found in pancreatic tumors [14]. These findings suggest that gingipains may exert pathogenic effects beyond the oral niche. Additionally, some studies have indicated that oral administration of Pg might alter the composition of the gut microbiota in healthy mice [12]. Pg commonly contains prophages of genetic diversity, and the genes they encode may alter the ecological potential and physiological functions of Pg, thereby potentially mediating its adaptation to the surrounding microbiota [15]. Therefore, whether gingipains contribute to the progression of intestinal inflammation by affecting the gut microbiota requires further investigation.

In this study, we observed that Pg inoculation induced dysbiosis of the gut microbiome in the mice, which triggered the increase of Th17 cells and the release of associated pro-inflammatory cytokines, consequently exacerbating intestinal inflammation. This inflammatory response was abolished by inhibiting Th17 activation. Cohabitation with healthy control mice facilitated gut microbiome restoration, conferring protection to the Pg-gavaged hosts against Th17 activation and inflammation. The key virulence factors of Pg, the gingipains, played a crucial role in gut microbiome dysbiosis and Th17-mediated immune response, as the sequential knockout of individual gingipains progressively ameliorated the phenotypes induced by the wild-type Pg, with the knockout of all three gingipains exhibiting the most significant mitigation of inflammation. Notably, immunization with inactivated Pg prevented Pg-induced gut microbiome dysbiosis, Th17 activation, and inflammation, suggesting potential intervention strategies. In summary, our study expands the understanding of how Pg may aggravate intestinal inflammation via modulation of the gut microbiome and subsequent immune dysregulation.

## Materials and methods

### Mice

Eight-week-old female C57BL/6 J mice free of specific pathogens were purchased from GemPharmatech Co., Ltd. (Nanjing, China). All animal procedures adhered strictly to the guidelines outlined in the National Research Council’s Guide for the Care and Use of Laboratory Animals and were approved by the Animal Care and Use Committee of Nanchang University (SYXK (Gan) 2021–0004). The mouse diet, acquired from Jiangsu XieTong Biotech Co., Ltd., consisted of a blend of corn, wheat, animal protein, amino acids, vitamins, and trace elements. Mice were housed in individually ventilated cage systems maintained at a temperature of 20± 2℃ and a relative humidity of 50 to 70%, following a 12:12-h light–dark cycle.

### Preparation and oral administration of Pg

Pg strain W83 was acquired from the Guangdong Microbial Culture Collection Center (GDMCC) and cultivated in an anaerobic chamber (Coy, Grass Lake, USA) with an atmosphere comprising 5% hydrogen, 5% carbon dioxide, and 90% nitrogen at 37 °C. The strain was cultured in Brain Heart Infusion (BHI) medium supplemented with hematin chloride (5 μg/mL) and vitamin K (1 μg/mL) under anaerobic conditions at 37 °C, maintaining the medium’s pH between 7.0 and 7.2 [16]. Bacterial purity was confirmed by Gram staining and phase contrast microscopy. The bacterial growth curve was determined to identify the logarithmic growth phase. A standard curve relating optical density (OD) to colony-forming units (CFUs) was established by measuring colony counts at various OD values. Based on the standard curve, 10^9^ CFU of Pg was collected during the logarithmic growth phase and centrifuged at 14,000 rpm for 10 min at 4 °C, followed by twice washing with PBS, and finally resuspended in a specific volume of PBS for further administration experiments.

### Pg treatment experiment

After a 1-week acclimatization period, the mice were randomly divided into four groups: the PBS group, the Pg group, the DSS group, and the DSS + Pg group. The DSS and DSS + Pg groups were treated with 2% DSS continuously for 7 days. Mice in the Pg and DSS + Pg groups received oral gavage of Pg at a total dose of 10⁹ CFU suspended in 200 μL of PBS per mouse daily for ten consecutive days. Mice in the PBS and DSS groups were gavaged with 200 μL of PBS without Pg.

### Th17 inhibition experiment

The DSS + Pg group mice received DSS treatment (days 0–7) and were orally gavaged with 10⁹ CFU of Pg suspended in 200 μL of PBS (days 0–10). The DSS + Pg + GSK805 group mice received DSS and Pg treatment concurrently and were also administered daily with 10 mg/kg of GSK805 (RORγt Inverse Agonist II, HY-12776, MedChemExpress) dissolved in DMSO (Sigma) via oral gavage in corn oil (days 0–10). The DSS + Pg + Anti-IL-17A group mice received DSS and Pg treatment concurrently and were intraperitoneally treated with 0.5 mg of monoclonal anti-IL-17A blocking antibody every 2 days for 10 days (days 0–10). These intervention groups were designed to evaluate the specific roles of Th17 cell expansion in Pg-exacerbated colitis and thus represent functional validation arms beyond the DSS + Pg model.

### Gingipain-knockout strains experiment

To investigate the role of gingipains in exacerbating intestinal inflammation by Pg, gingipain knockout strains were employed. All mice were randomly divided into seven groups after 5 days of antibiotic treatment (ampicillin 0.2 g L^−1^, metronidazole 0.2 g L^−1^, neomycin 0.2 g L^−1^, vancomycin 0.1 g L^−1^) following previous reports [17, 18]. The PBS group received a regular diet and was orally gavaged with 200 μL of PBS (days − 5 to 10). The DSS group received 2% DSS treatment for 7 days (days 0–7) and was orally gavaged with 200 μL of PBS (days − 5 to 10). The DSS + WT, DSS + **∆**Kgp, DSS + **∆**RgpA/B, and DSS + **∆**KRAB groups all received the same DSS treatment (days 0–7) but were orally gavaged with 10⁹ CFU of WT Pg, **∆**Kgp, **∆**RgpA/B, or **∆**KRAB, respectively, each suspended in 200 μL of PBS (days − 5 to 10).

### Co-housing experiment

A co-housing (Co) experiment was conducted to investigate whether mice infected with Pg would show reduced intestinal inflammation after co-housing with healthy mice control. Following a 1-week acclimatization period, all mice underwent a 5-day antibiotic treatment regimen (ampicillin 0.2 g L^−1^, metronidazole 0.2 g L^−1^, neomycin 0.2 g L^−1^, and vancomycin 0.1 g L^−1^) before randomly assigned to four groups: PBS, DSS + Pg, Co-PBS, and Co-DSS + Pg. Mice in the Co-PBS and Co-DSS + Pg groups were housed together in the same cage to facilitate microbiota transfer. Subsequently, mice in the DSS + Pg and Co-DSS + Pg groups were orally gavaged with 200 μL of PBS containing 10⁹ CFU of Pg daily for 14 consecutive days. Starting on day 0, 2% DSS was administered for 7 days to induce colitis. Mice in the PBS and Co-PBS groups were gavaged with 200 μL of PBS without Pg during the same period.

### Immunization experiment

A total of 10^6^ CFU of Pg and 10^8^ CFU of Pg were heat-inactivated and administered to mice via subcutaneous injection. The control group was injected with PBS. Immunizations were performed every 2 weeks for a total of four injections. Following the completion of immunization, blood was collected from the tail vein of the mice to measure Pg-specific antibody levels. Subsequently, mice were treated with 2% DSS for seven consecutive days. During this period, mice were also orally gavaged with 200 μL of PBS containing 10⁹ CFU of Pg for ten consecutive days.

### Histology activity index, disease activity index

Fresh colon samples were fixed in 4% paraformaldehyde and embedded in paraffin. The paraffin-embedded colon tissue sections were stained with hematoxylin and eosin (HE). Colonic histopathology was scored according to the previously established tissue scoring system, as follows: histopathological changes in crypt structure (0–4 scale), the extent of inflammatory infiltration (0–4 scale) [19, 20]. Disease activity index (DAI) is commonly used to assess the severity of intestinal inflammation, scored based on mouse weight loss, fecal consistency, and fecal occult blood [21].

### DNA extraction and 16S rRNA amplicon sequencing

Microbial DNA was extracted from fecal samples using the Tiangen-DNA Stool Kit (TIANGEN, Beijing, China). The concentration of DNA was assessed using Nanodrop (Thermo Fisher Scientific, USA). The V4 region of bacterial 16S rDNA was specifically targeted with barcoded primers (515F, 5′-GTGCCAGCMGCCGCGGTAA-3′, 806R, 5′-GGACTACHVGGGTWTCTAAT-3′) for sequencing, which was carried out using an Illumina NovaSeq platform at Novogene, Co., Ltd. (Beijing, China). An equal amount of DNA was used to prepare sequencing libraries. Sequencing libraries were prepared with the TruSeq® DNA PCR-Free Sample Preparation Kit (San Diego, CA, USA), following the manufacturer’s guidelines. Library quality was assessed using a Qubit 2.0 Fluorometer (Thermo Fisher Scientific, USA) before sequencing on the Illumina NovaSeq platform (Illumina, San Diego, USA). Quality control measures, including negative controls for DNA extraction and PCR amplification, as well as a mock community, were incorporated into each HiSeq run.

### Microbiota data analysis

The raw paired-end reads were joined using FLASH [22]. The sequencing reads were subjected to quality filtering for the removal of low-quality sequences using the Fastp program [23]. Deblur was utilized for generating high-quality amplicon sequence variant (ASV) Table [24]. The taxonomic classification was performed using the SILVA database and QIIME2 (quantitative insights into microbial ecology 2, version 2022.2) q2 feature-classifier plugin [25]. Alpha and beta diversity metrics were calculated using the QIIME2 q2-diversity plugin. Principal coordinates analysis (PCoA) plots were visualized using EMPeror [26]. The PERMANOVA test was conducted to assess the structural discrepancy of the bacterial community. Differential bacterial genera between groups were identified using linear discriminant analysis (LDA) effect size (LEfSe) [27], with only bacterial taxa possessing LDA scores exceeding the threshold of 2.0 being displayed. MaasLin2 was also used to perform differential abundance analysis of microbial genera with an FDR threshold of 0.05 [28]. Spearman correlation was calculated between the concentrations of inflammatory indices and bacterial taxa of differential abundances via the stats package. Microbial traits were predicted using BugBase, with a threshold of 0.1% for low-abundance taxa and normalization to sequencing depth 40,000, using the tool’s default settings [29].

### Construction of Pg mutant ∆kgp (single-gene knockout), ∆RgpA/B (double-gene knockout), and ∆KRAB (triple-gene knockout)

To construct gingipain-inactivated mutants, plasmids listed in Supplementary Table 1 were generated [30, 31] using primers listed in Supplementary Table 2. 1-kb or 2.5-kb fragments of the upstream and downstream sequences of the kgp, rgpA, or rgpB were amplified from the chromosomal DNA of Pg W83. For gene knockout and mutant selection, three resistance cassettes (i.e., ermF, tetQ, and cat) were used and amplified with primers that contained overlapping nucleotides with the upstream and downstream fragments, respectively. For knockout of kgp or rgpA, the cat cassette was assembled with the upstream fragment, the downstream fragment, and the pUC origin of replication to give pKN-kgp-cat and pKN-rgpA-cat. For the double and triple mutants, the tetQ and ermF cassettes were used as the second and third selection markers, assembled with the upstream and downstream fragments of rgpB or kgp, and cloned into pUC19 at SacI and HindIII sites to give pUC19-rgpB-tetQ and pUC19-kgp-ermF. Using these plasmids as the template, the DNA fragment for gene knockout was amplified with the forward primer of the upstream fragment and the reverse primer of the downstream fragment and transformed into Pg by electroporation as previously described [32]. Briefly, 10 mL of culture in the exponential growth phase was bathed on ice for 15 min and harvested by centrifugation at 4℃. The pelleted cells were washed three times with pre-cooled electroporation buffer (10% glycerol, 1 mM MgCl_2_), resuspended in 100 μL electroporation buffer, and mixed with 2 μg DNA fragment, followed by electroporation at 2500 V using Gene Pulser (Bio-Rad). The cell suspension after electroporation was immediately transferred to 1-mL fresh medium and incubated at 37℃ for approximately 16 h. The cells were pelleted by centrifugation, resuspended in 100 μL BHI broth, and plated on BHI agar containing appropriate antibiotics (e.g., 10 μg/mL chloramphenicol, 5 μg/mL erythromycin, or 3 μg/mL tetracycline). The plates were incubated at 37℃ for 7–10 days until colonies appeared. The correct mutants were confirmed by PCR and DNA sequencing. Primers used for the identification of Pg mutant strains are listed in Supplementary Table 2.

### Quantification of Pg in the Gut with qPCR

Colonic contents and colonic tissues were collected 12 h after the oral administration of Pg. Microbial DNA was then extracted using the QiAamp PowerFecal Pro DNA Kit (Qiagen, Hilden, Germany). Quantitative real-time PCR (qPCR) was performed using the CFX 96 Real-Time PCR Detection System, and relative abundance was calculated with the ΔCt method [14]. Universal 16S rRNA was amplified using the forward primer 5′-ACTCCTACGGGAGGCAGCAGT-3′ and the reverse primer 5′-ATTACCGCGGCTGCTGGC-3′. For the amplification of Pg16S rRNA, the forward primer 5′-AGGCAGCTTGCCATACTGCG-3′ and the reverse primer 5′-ACTGTTAGCAACTACCGATGT-3′ were used [15].

### Flow cytometry

Fresh mouse spleens were collected and ground with a 2-mL syringe needle in a pre-cooled culture dish until the splenic capsules were completely ruptured. Then, the resulting suspension was passed through a 70-μm cell strainer to remove connective tissue. The cell suspension was transferred into a 15-mL centrifuge tube, followed by centrifugation at 1500 rpm for 8 min. Then, the supernatant was discarded, followed by the addition of 4 mL of red cell lysate, and the mixture was shaken and allowed to stand at room temperature for 5 min until no visible red residue remained. Subsequently, the remaining pellet was washed with precooled RPMI 1640 medium, followed by another centrifugation under the same conditions. Finally, the pellet was resuspended in pre-cooled RPMI 1640 medium supplemented with 10% fetal bovine serum to prepare a single-cell suspension.

Approximately 10^6^ splenic lymphocytes were incubated with Cell Activation Cocktail (with Brefeldin A) (BD Biosciences, USA) in a CO_2_ incubator for 6 h. After incubation, the cells were stained with Fixable Viability Stain 510 for 30 min at room temperature (RT) in the dark. Following this, BB700-labeled anti-CD4 antibodies and BB515-labeled anti-CD25 antibodies (BD Biosciences, USA) were added for staining at 4 °C for 30 min in the dark. The cells were then fixed and permeabilized using Fixation/Permeabilization working solution and Permeabilization Buffer (BD Biosciences, USA) for 20 min at RT in the dark. Intracellular staining was subsequently performed with PE-labeled anti-IL-17A and APC-labeled anti-Foxp3 antibodies (BD Biosciences, USA) at 4 °C for 30 min. Finally, the cells were resuspended in PBS containing 2% fetal bovine serum and analyzed using flow cytometry.

Cell populations were analyzed using FlowJo software (version 10.6.3, BD, USA). Lymphocytes were initially identified based on forward scatter area (FSC-A) and side scatter area (SSC-A) parameters, representing cell size and granularity, respectively. Cellular debris and doublets were excluded by gating on FSC-H and FSC-A. Within the resulting singlet population, live cells were selected by excluding Fixable Viability Stain 510-positive events. For Th17 cell analysis, CD4⁺ T cells were first gated, followed by identification of IL-17A⁺ cells on a univariate axis, with Th17 cells defined as CD4⁺IL-17A⁺. For Treg analysis, CD4⁺ T cells were similarly gated, then biaxial CD25^+^Foxp3^+^, with Treg cells defined as CD4⁺CD25⁺Foxp3⁺.

### Enzyme-linked immunosorbent assay

Colon tissues were weighed and homogenized in 900 mL of PBS using ultrasonic disruption, followed by centrifugation at 3000 rpm for 10 min to obtain the tissue homogenate. Cytokine levels of IL-6 (#88–7064-22, Thermo Fisher Scientific), IL-17A (#88–7371-22, Thermo Fisher Scientific), IL-23 (#88–7230-22, Thermo Fisher Scientific), and IL-22 (#88–7422-22, Thermo Fisher Scientific) were quantified using ELISA according to the manufacturer’s protocol. In brief, capture antibodies were coated onto microplate wells and incubated overnight at 4 °C. After blocking, samples were added to the wells and incubated for 2 h at room temperature, followed by incubation with detection antibodies for 1 h. Subsequently, avidin-conjugated horseradish peroxidase was added and incubated for 30 min. Afterward, the TMB substrate solution was added and incubated for 15 min, followed by the addition of a stop solution. Absorbance was measured at 450 nm using a microplate reader, and cytokine concentrations were determined from the standard curve.

### Analysis of gene expression in the colon

Total RNA was extracted from colonic samples using the RNAiso Plus kit (Takara, Beijing, China). The extracted RNA (1 μL) was reverse transcribed using the PrimeScript™ RT reagent Kit (Takara, Beijing, China). Gene expression analysis was conducted using the CFX Connect real-time PCR detection system (Bio-Rad Laboratories, Singapore) with TB Green® Premix Ex Taq™ II (Takara, Beijing, China). GAPDH served as an internal reference control, and the relative gene expression levels were determined using the 2^−ΔΔ^Ct method, as previously described [33, 34]. Primers used in this section are listed in Supplementary Table 3.

### Immunofluorescence analysis

Frozen colon tissues were sectioned at 8 μm thickness and fixed in 4% paraformaldehyde for 15 min at room temperature. After three washes with PBS, sections were permeabilized with 0.3% Triton X-100 for 10 min and blocked with 10% normal goat serum for 30 min. Slides were then incubated overnight at 4 °C with an anti–IL-17A primary antibody (BOSTER Biological Technology Co., Ltd). After PBS washing, sections were incubated with fluorescently labeled secondary antibodies (Jackson ImmunoResearch Company) for 1 h at room temperature, followed by nuclear counterstaining with DAPI. Images were captured using a fluorescence microscope, and quantitative analyses were conducted using ImageJ.

### Statistical analysis

The statistical analysis was performed using Prism 8.2.1 (GraphPad Software, CA). Data were represented as means ± SEM. Data normality was assessed prior to statistical testing. Differences between two groups were evaluated using the Student’s *t*-test for normally distributed data, or the Mann–Whitney *U* test otherwise. For comparisons among more than two groups, one-way ANOVA was used for normally distributed data, or the Kruskal–Wallis test otherwise, followed by Tukey’s post hoc test where appropriate.

## Results

### Pg exacerbated intestinal inflammation

**Fig. 1:**
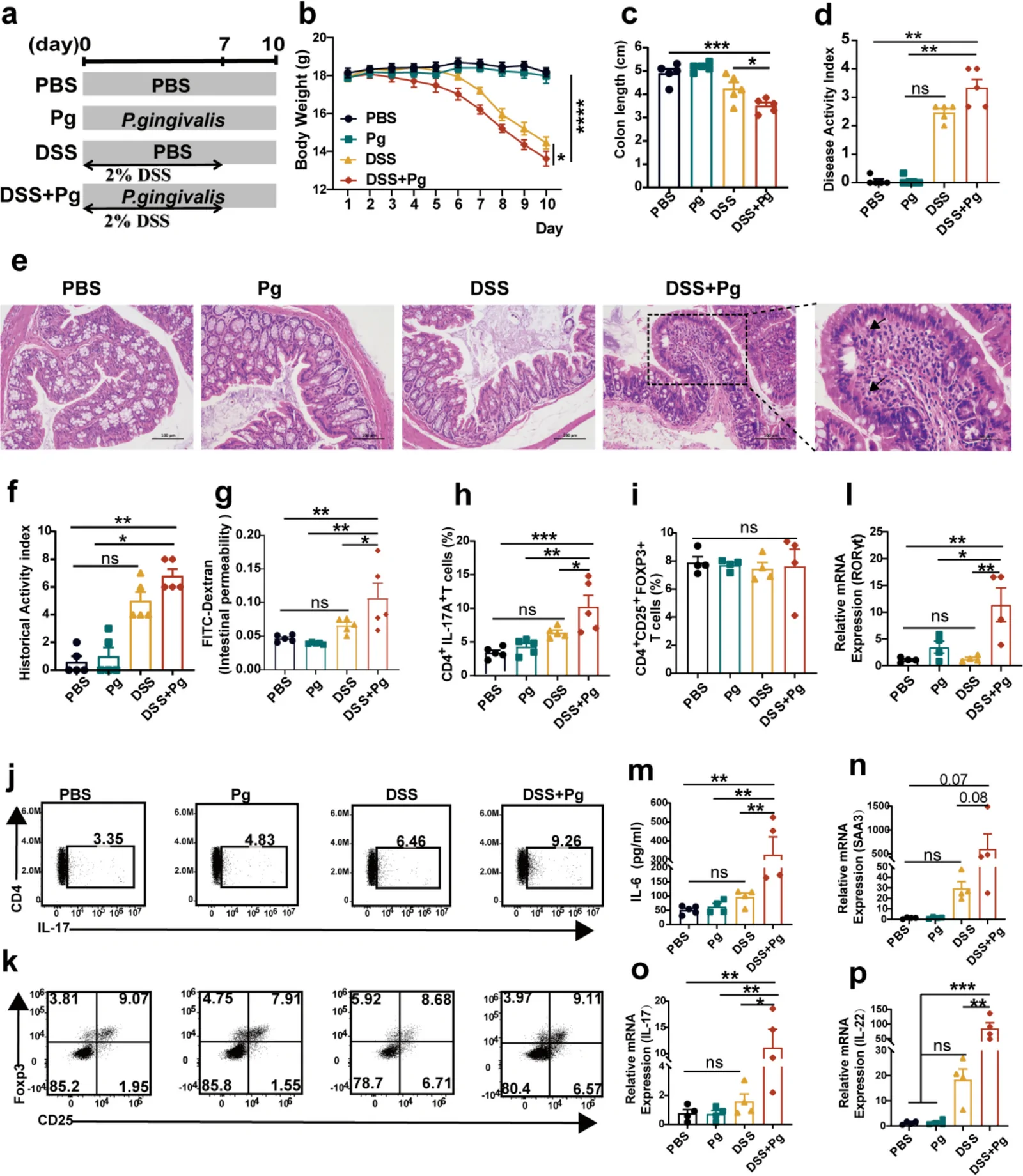
Pg exacerbated intestinal inflammation. **a** The Pg treatment experimental design. **b** Body weight (*n* = 5 per group). **c** Colon length (*n* = 5 per group). **d** Disease activity index (DAI) (*n* = 5 per group). **e** Representative images of H&E-stained colonic sections and **f** histological scores of colons. Scale bar = 100 μm. The magnified inset shows the DSS + Pg group (scale bar = 50 μm). Black arrows indicate neutrophil infiltration (*n* = 5 per group). **g** Intestinal permeability (*n* = 5 per group). **h**–**k** The proportions of IL-17^+^ Th17 cells (*n* = 5 per group) and Foxp3.^+^ Treg cells (*n* = 4 per group) within the spleens were detected by flow cytometry. **l** The relative mRNA expressions of RORγt. **m** Concentrations of serum IL-6 measured by ELISA (*n* = 4 per group). **n**–**p** The relative mRNA expressions of IL-17 (**o**), SAA3 (**n**), and IL-22 (**p**) in colon tissues (*n* = 4 per group). The data were presented as the mean ± SEM. The data in Fig. 1 d and Fig. 1f did not pass the normality test and were therefore analyzed using the Kruskal–Wallis test, while the other data were analyzed using one-way ANOVA with Tukey’s test. *p ≤ 0.05, **p ≤ 0.01, ***p ≤ 0.001

To assess the impact of Pg in the gut, we administered a daily dose of 10^9^ CFU of Pg for 10 days with or without an oral gavage of 2% DSS to mice for 7 days (Fig. 1a). Mice treated with DSS + Pg exhibited aggravated inflammation compared to mice treated with PBS or DSS alone, as evident from body weight loss, colon shortening, DAI, and histology activity index (HAI) (Fig. 1b–f). However, Pg alone did not induce observable changes compared to the control mice. Additionally, gut barrier function was compromised in DSS + Pg mice with increased intestinal permeability (Fig. 1g). Immunofluorescence analysis showed a significant reduction in ZO-1 levels in DSS + Pg mice (Supplementary Fig. 1a–b), although E-Cadherin, Occludin, and MUC2 showed no differences among the groups (Supplementary Fig. 1c–f). Given that CD4^+^ T lymphocytes are involved in oral microbiota-mediated systemic diseases [35], we measured immune cell populations with flow cytometry and observed a higher proportion of IL-17^+^ Th17 cells in the DSS + Pg group and no statistically significant difference in Foxp3 + Treg cell proportions (Fig. 1h–k). qPCR analysis revealed that the relative mRNA expressions of RORγt—a key transcription factor for Th17 cell differentiation[36]—were elevated in the colonic tissues of mice in the DSS + Pg group (Fig. 1l). Following this, we investigated the pertinent cytokines in the Th17 cells pathway. We observed a notable elevation of the cytokine upstream of Th17 cells, IL-6, in the DSS + Pg group (Fig. 1m), whereas TGF-β exhibited no significant alterations (Supplementary Fig. 1g). Given the potential of serum amyloid A proteins (SAA) to promote the differentiation of pro-inflammatory Th17 cells in conjunction with IL-6 [36], we measured their expression levels and observed a substantial increase in the expressions of SAA3 in the DSS + Pg group (Fig. 1n), whereas SAA1 and SAA2 did not change significantly (Supplementary Fig. 1 h–i). Furthermore, the cytokines downstream of Th17 cells, IL-17 and IL-22, were markedly elevated in the DSS + Pg group (Fig. 1o–p). These findings suggest a potential link between Pg exposure, aggravated intestinal inflammation, and increased Th17-associated immune responses in mice.

### Th17-mediated immune responses were involved in intestinal inflammation aggravated by Pg

**Fig. 2:**
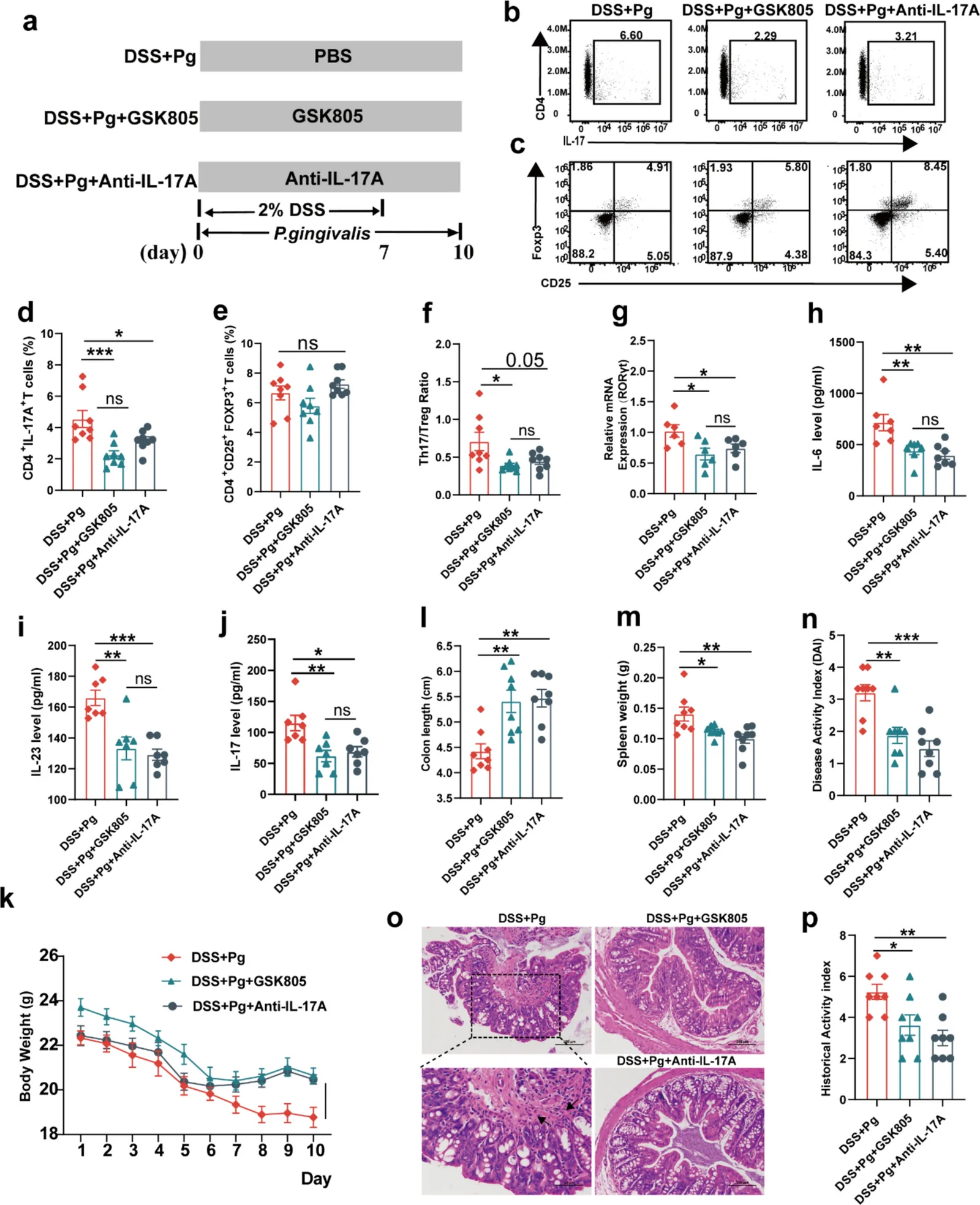
Th17-mediated immune responses were involved in intestinal inflammation aggravated by Pg. **a** Th17 inhibition experimental design. **b**–**f** The proportions of IL-17^+^ Th17 cells and Foxp3^+^ Treg cells and their ratio among total CD4.^+^ T cells within the spleens were detected by flow cytometry (*n* = 8 per group). **g** The relative mRNA expressions of RORγt (*n* = 6 per group). **h**–**j** Concentrations of IL-6, IL-17, and IL-23 measured by ELISA (*n* = 7 per group). **k** Body weight. **l** Colon length. **m** Spleen weight. **n** DAI. **o** H&E-stained colon sections and **p** histological scores of colons (*n* = 8 per group). Scale bar = 100 μm. The magnified inset shows the DSS + Pg group (scale bar = 50 μm). Black arrows indicate neutrophil infiltration. The data were presented as the mean ± SEM and *p* values were assessed by one-way ANOVA with Tukey’s test. *p ≤ 0.05, **p ≤ 0.01, ***p ≤ 0.001

To investigate the role of Th17 cells in exacerbating intestinal inflammation caused by Pg, we inhibited the expansion of Th17 cells with GSK805 (RORγt Inverse Agonist II), or monoclonal anti-IL-17A blocking antibody in the mice treated with DSS + Pg (Fig. 2a). As shown in Fig. 2b–f, the increase of Th17 cells and the Th17/Treg ratio were significantly lower in GSK805 or anti-IL-17A antibody treatment compared to the DSS + Pg group. This observation was subsequently validated by decreased RORγt expression levels (Fig. 2g). The GSK805 and anti-IL-17A antibody also significantly reduced the levels of cytokines associated with Th17 cells in mouse colon tissues, including IL-6, IL-17, and IL-23 (Fig. 2h–j). Notably, GSK805 or anti-IL-17A antibody significantly alleviated the body weight loss, colon shortening, increased spleen weight, DAI, and HAI (Fig. 2k–p). QPCR results also demonstrated a reduction in the levels of SAA2 and SAA3 in colon tissues following treatment with anti-IL-17A antibody (Supplementary Fig. 2a–c). In addition, we utilized 16S rRNA amplicon sequencing to evaluate the impact of Th17 inhibitors on the composition of the gut microbiota in mice. The GSK805 and Anti-IL-17 antibody groups showed no significant differences in alpha diversity (Supplementary Fig. 3a–b), beta diversity (Supplementary Fig. 3c–d), or microbiota composition at both the phylum and genus levels (Supplementary Fig. 3e–f) compared to the DSS + Pg group. Taken together, these results suggest that inhibition of Th17 responses may help mitigate the Pg-induced pro-inflammatory phenotype, indicating a potential mediating role of Th17 cells in this pathological process.

### Gingipain knockout mitigated Pg-aggravated intestinal inflammation

**Fig. 3:**
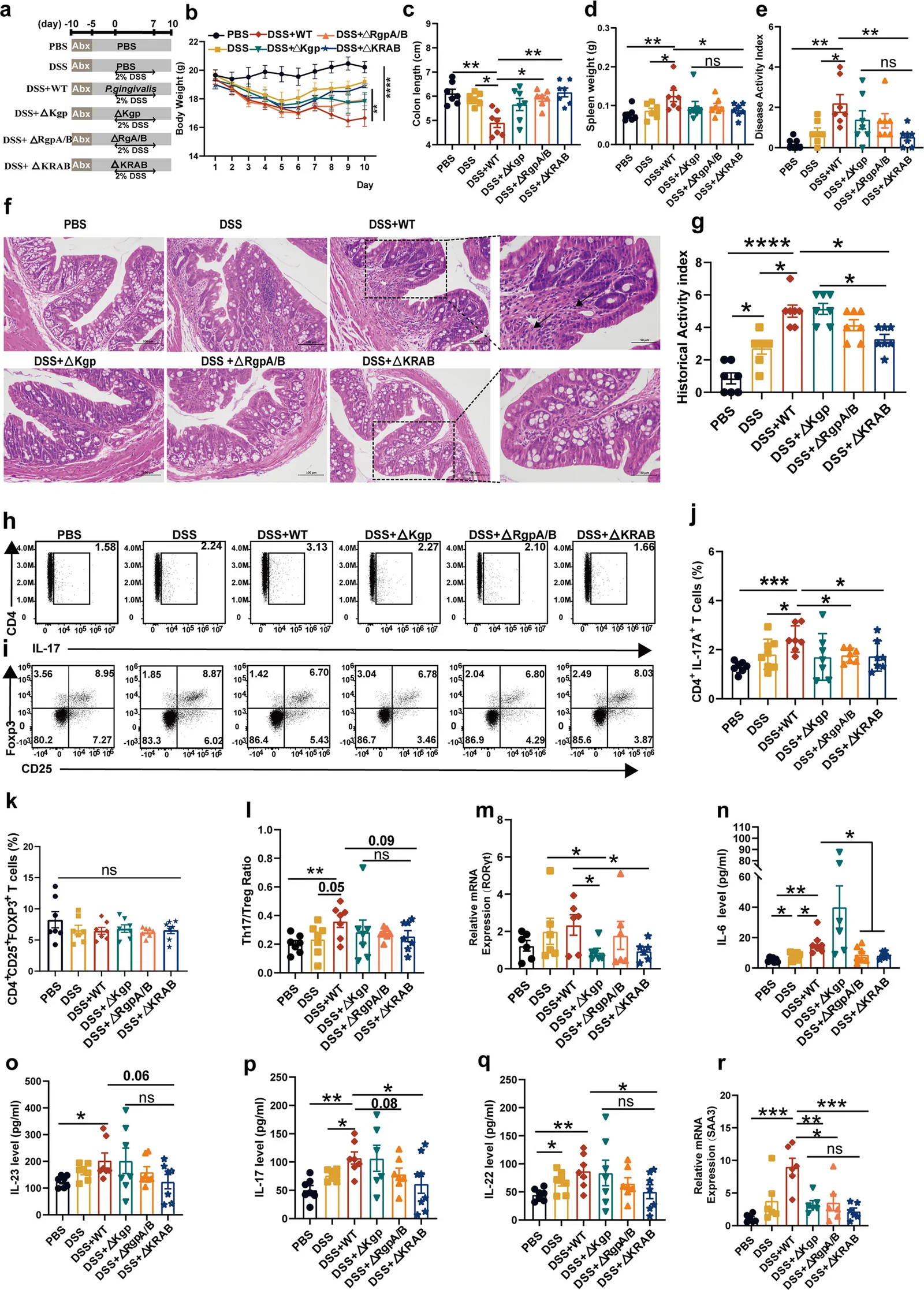
Gingipain knockout mitigated Pg-aggravated intestinal inflammation. **a** The gingipain-knockout strains experimental design. **b** Body weight (*n* = 7 per group). **c** Colon length (*n* = 7 per group). **d** Spleen weight (*n* = 7 per group). **e** DAI (*n* = 7 per group). **f** Representative images of H&E-stained colonic sections. **g** Histological scores of colons. Scale bar = 100 μm. The magnified inset (scale bar = 50 μm) shows the DSS + Pg group. Black arrows indicate neutrophil infiltration (*n* = 7 per group). **h**–**l** The proportions of IL-17^+^ Th17 cells and Foxp3^+^ Treg cells and their ratio among total CD4.^+^ T cells within the spleens were detected by flow cytometry (*n* = 7 per group). **m** The relative mRNA expressions of RORγt (*n* = 6 per group). **n**–**q** Concentrations of IL-6, IL-17, IL-23, and IL-22 measured by ELISA. **r** The relative mRNA expressions of SAA3 (*n* = 6 per group). The data were presented as the mean ± SEM and *p* values were assessed by one-way ANOVA with Tukey’s test. *p ≤ 0.05, **p ≤ 0.01, ***p ≤ 0.001

To investigate the role of Pg virulence factors in exacerbating intestinal inflammation, we gavage mice with gingipain-knockout strains, including *kgp* knockout (**∆**Kgp), *rgpA* and *rgpB* double knockout (**∆**RgpA/B), and triple knockout of both Kgp and RgpA/B (**∆**KRAB) (Fig. 3a). The knockout and validation process for the mutant strains are provided in Supplementary Fig. 4. Compared to the wild-type Pg, the DSS + **∆**RgpA/B, DSS + ∆Kgp, and DSS + **∆**KRAB groups exhibited varying degrees of alleviation in the intestinal inflammation (Fig. 3b–g), with the triple knockout strains displaying the most pronounced alleviation and no difference from DSS treatment alone. A significant decrease in the proportion of Th17 cells was observed in the DSS + **∆**RgpA/B and DSS + **∆**KRAB groups compared to the DSS + WT group. Consistent with these findings, immunofluorescence staining revealed a marked increase in IL-17–positive cells in the DSS + WT group in the colon, whereas this elevation was noticeably attenuated in the DSS + **∆**KRAB groups (Supplementary Fig. 5a–b). The ratio of Th17/Treg cells in the DSS + **∆**KRAB group also showed a significant decrease, although the proportion of Treg cells did not show a significant difference (Fig. 3h–l). These observations aligned with the downregulation of RORγt and SAA3 gene expressions (Fig. 3m and 3r). Furthermore, Th17-associated cytokine levels followed a similar alleviation trend. The DSS + ΔKgp group showed no significant changes, the DSS + ΔRgpA/B group exhibited alleviation in IL-6 and IL-17 levels, and the DSS + ΔKRAB group displayed the most pronounced alleviation across multiple cytokines, including IL-6, IL-17, IL-23, and IL-22 (Fig. 3n–q). This indicates that the gingipains RgpA/B and Kgp differentially contribute to the increase of Th17 cells and cytokine production, with a cumulative effect observed upon their combined knockout. The expression levels of tight junction proteins (ZO-1 and Occludin) and the mucus-related protein MUC2 showed no significant changes; similarly, no significant differences were observed in SAA1 and SAA2 levels (Supplementary Fig. 6a–e). Taken together, these findings suggest that gingipains may contribute to Pg-induced the increase of Th17 cells and the exacerbation of intestinal inflammation observed in Pg-treated mice.

### Pg induced the gut microbiota dysbiosis in a gingipain-dependent manner

**Fig. 4:**
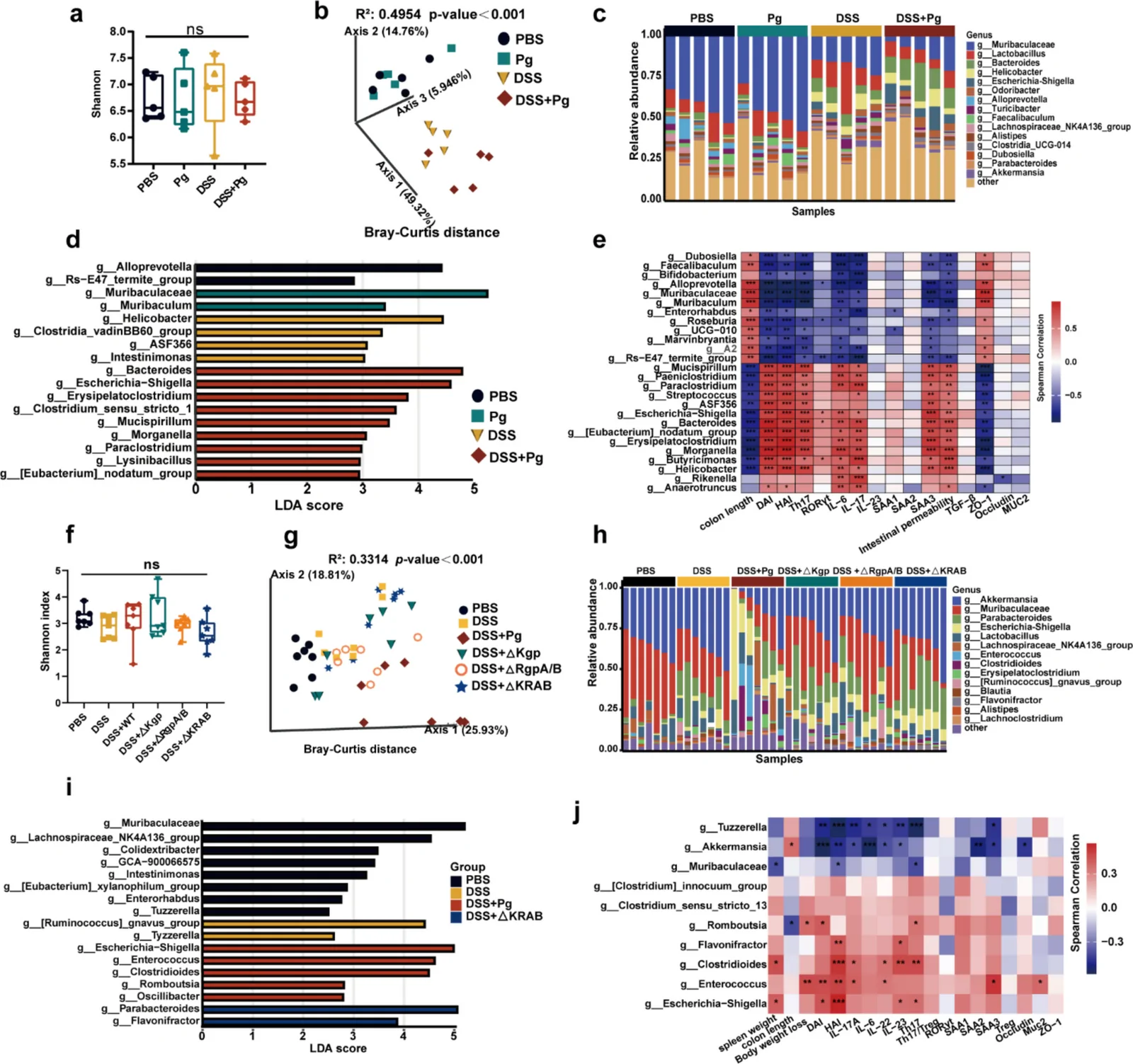
Pg induced the gut microbiota dysbiosis in a gingipain-dependent manner. **a** Alpha diversity analysis of the gut microbiota from the Pg treatment experiment measured with Shannon index. **b** Bray–Curtis distance-based PCoA of the gut microbiota from the Pg treatment experiment. **c** The microbiota composition at the genus level. **d** Bacterial taxa of differential abundance among groups with the linear discriminant analysis (LDA) scores derived from LEfSe analysis (LDA > 2.0) (*n* = 5 per group, two cages). **e** Spearman correlation between gut microbiota and pathological indicators in the Pg treatment experiment. *p ≤ 0.05, **p ≤ 0.01, ***p ≤ 0.001. **f** Alpha diversity analysis of the gut microbiota from the gingipain-knockout strains experiments measured with the Shannon index. **g** Bray–Curtis distance-based PCoA of the gut microbiota from the gingipain-knockout strains experiment. **h** The microbiota composition at the genus level in the gingipain-knockout strains experiment. **i** Bacterial taxa of differential abundance among groups with the LDA scores derived from LEfSe analysis (LDA > 2.0) in the gingipain-knockout strains experiment (*n* = 7 per group, two cages). **j** Spearman correlation between gut microbiota and pathological indicators in the gingipain-knockout strains experiment. *p ≤ 0.05, **p ≤ 0.01, ***p ≤ 0.001

The mouse fecal samples of all the experimental groups before and after treatments were characterized with 16S rRNA amplicon sequencing to assess the effect of Pg on the gut microbiota. The analysis of background microbiota prior to treatment showed no significant differences across different groups (Supplementary Fig. 7), eliminating the potential confounding effects of background microbiota. At the end of 10-day treatment, DSS or Pg did not significantly affect alpha diversity (Fig. 4a, Supplementary Fig. 8b). However, beta diversity analysis based on weighted UniFrac distance (Supplementary Fig. 8c) and Bray–Curtis (Fig. 4b) revealed that microbial clustering in the DSS + Pg group was significantly distinct from the PBS, Pg, and DSS groups (PERMANOVA, *p* < 0.001). Notably, while DSS treatment induced moderate gut microbiota dysbiosis, the administration of Pg further exacerbated these alterations. The DSS + Pg group showed more pronounced shifts in microbial composition compared to the DSS group, with significant separation observed between the two groups (PERMANOVA, *p* < 0.001). At the genus level, MaAsLin2 analysis revealed that Pg administration upon DSS further affected the relative abundance of specific microbial genera. The *Alloprevotella* and *Muribaculum* were significantly lower in the DSS + Pg group compared to the other treatment groups (*p* < 0.05), while *Bacteroides* and *Escherichia-Shigella* showed a marked increase (Fig. 4c, Supplementary Fig. 9). Consistently, LEfSe analysis revealed that *Bacteroides*, *Morganella*, and *Escherichia-Shigella* were enriched in the DSS + Pg group (Fig. 4d). These genera enriched in the DSS + Pg group exhibited a positive Spearman correlation with Th17 cell proportions, DAI, HAI, and other inflammatory markers, while those with reduced abundance, including *Alloprevotella*, *Muribaculum*, and *Faecalibaculum*, showed a negative Spearman correlation (Fig. 4e).

We also assessed the impact of gingipain-knockout strains on gut microbiota. The α-diversity analysis using the Shannon index was similar among the groups (Fig. 4f), although Faith’s PD and Observed ASVs showed a decrease in the DSS + **∆**RgpA/B and DSS + **∆**KRAB groups compared to the control group (Supplementary Fig. 10b–c). PCoA revealed that the gut microbiota composition of the gingipain knockout groups clustered more closely with the control group compared to the wild-type Pg group (Fig. 4g, Supplementary Fig. 10 d). At the genus level, the reduction of *Akkermansia* induced by DSS + Pg treatment was less pronounced in the DSS + ΔKgp, DSS + ΔRgpA/B, and DSS + ΔKRAB groups (*p* < 0.05). Similarly, the elevations of *Escherichia-Shigella*, *Lactobacillus*, and *Enterococcus* were also attenuated to varying degrees in these knockout groups (*p* < 0.05). Notably, the DSS + **∆**KRAB group showed the most significant restoring effect (Fig. 4h–i). Spearman correlation analysis showed a significant negative correlation between the genera alleviated by gingipain-deficient isogenic strains (e.g., *Akkermansia*, *Enterococcus*) (Supplementary Fig. 10f) and inflammatory markers such as Th17 and DAI (Fig. 4j). Microbial traits predicted by Bugbase showed a significant decrease in mobile element-containing, facultative anaerobic, and Gram-negative bacteria in the gut microbiota treated with gingipain-null isogenic strains compared to those treated with the wild-type Pg (Supplementary Fig. 11). These findings indicated that gingipains may play critical roles in Pg-induced gut microbiota dysbiosis.

Importantly, we measured the levels of Pg in the gut using qPCR. The results showed that the its abundances in the mice gavaged Pg alone were not statistically different from the control mice. In contrast, the DSS + Pg group exhibited significantly elevated Pg levels compared to both the PBS and DSS groups, suggesting that Pg might gain a competitive advantage in the gut with inflammation, facilitating its persistence (Supplementary Fig. 12a). Additionally, no significant differences in abundance were observed between the knockout and the WT strains in the colonic tissues or contents of the gavaged mice (Supplementary Fig. 12b–c), indicating that the alleviation of gut microbiota dysbiosis may not be due to a weakened persistence of Pg resulting from gingipain knockout.

### Co-housing alleviated Pg-induced gut microbiota dysbiosis and intestinal inflammation

**Fig. 5:**
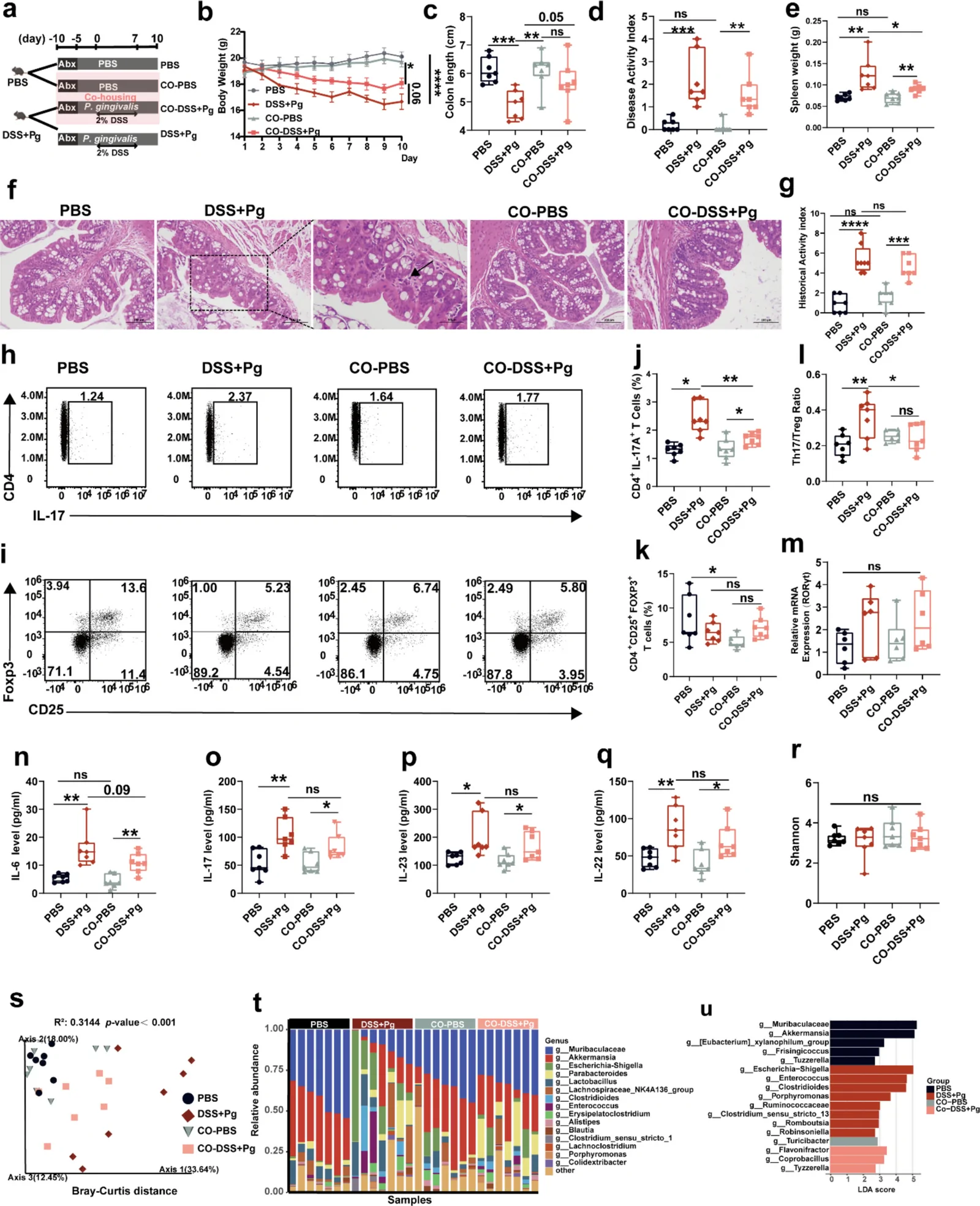
Gut microbiota dysbiosis mediated Pg-aggravated intestinal inflammation. **a** The co-housing experimental design. **b** Body weight (*n* = 7 per group). **c** Colon length. **d** DAI. **e** Spleen weight. **f** H&E stained colon sections and **g** histological scores of the colon (*n* = 7 per group). Scale bar = 100 μm. The magnified inset (scale bar = 50 μm) shows the DSS + Pg group. Black arrows indicate neutrophil infiltration. **h**–**l** The proportions of IL-17^+^ Th17 cells and Foxp3^+^ Treg cells and their ratio among total CD4.^+^ T cells within the spleens were detected by flow cytometry (*n* = 7 per group). **m** The relative mRNA expressions of RORγt (*n* = 6 per group). **n**–**q** Concentrations of IL-6, IL-17, IL-23, and IL-22 measured by ELISA (*n* = 7 per group). **r** The Shannon index. **s** PCoA based on Bray–Curtis distance of fecal microbiota. **t** The microbiota composition at the genus level. **u** Bacterial taxa of differential abundance among groups with the LDA scores derived from LEfSe analysis (LDA > 2.0) (*n* = 7 per group, two cages). The data were presented as the mean ± SEM and *p* values were assessed by one-way ANOVA with Tukey’s test. *p ≤ 0.05, **p ≤ 0.01, ***p ≤ 0.001

To investigate whether gut microbiota alterations could mediate Pg-aggravated intestinal inflammation, we co-housed mice treated with DSS + Pg and PBS to remodel the gut microbiota (Fig. 5a). The co-housing experiment leverages the coprophagic and grooming behaviors of mice to facilitate microbiota sharing among individuals within the same cage, aiming to determine whether changes in gut microbiota mediate related diseases [37, 38]. As shown in Fig. 5b–g, co-housing partially alleviated the intestinal inflammation induced by DSS + Pg treatment, as measured by body weight loss, colon length, DAI, spleen weight, and HAI. The increase of Th17 cells and the Th17/Treg cell ratio were significantly reduced in the Co-DSS + Pg group compared to the DSS + Pg group (Fig. 5h–l). This corroborated with the decreased mRNA expression levels of RORγt in the Co-DSS + Pg group (Fig. 5m). Similarly, co-housing significantly reduced the levels of Th17 pathway-related cytokines, including IL-6, IL-17, IL-22, and IL-23 (Fig. 5n–q) as well as SAA3 (Supplementary Fig. 13f), although ZO-1, Occludin, MUC2, SAA1, and SAA2 did not exhibit significant changes (Supplementary Fig. 13a–e). To characterize the gut microbiota during the co-housing, we performed a comparative analysis with 16S rRNA amplicon sequencing. Co-housing did not affect the alpha diversity among the groups (Fig. 5r, Supplementary Fig. 14a–b). The PCoA analysis revealed that microbial clustering in the Co-DSS + Pg group was positioned between the PBS and DSS + Pg groups (PERMANOVA, *p* < 0.001), suggesting that the microbiota underwent remodeling during the co-housing process (Fig. 5s, Supplementary Fig. 14c). Specifically, co-housing notably alleviated the reduced levels of *Akkermansia* and Muribaculaceae and the increased level of *Escherichia-Shigella* caused by DSS + Pg (Fig. 5t), which was concordance with differential abundance analysis (Fig. 5u). These findings, together with the minimal impact of Th17 inhibition on the gut microbiota, suggest that Pg-associated gut microbiota dysbiosis may be linked to enhanced Th17 responses and the worsening of intestinal inflammation.

### Immunization with inactivated Pg prevented Pg-aggravated intestinal inflammation

**Fig. 6:**
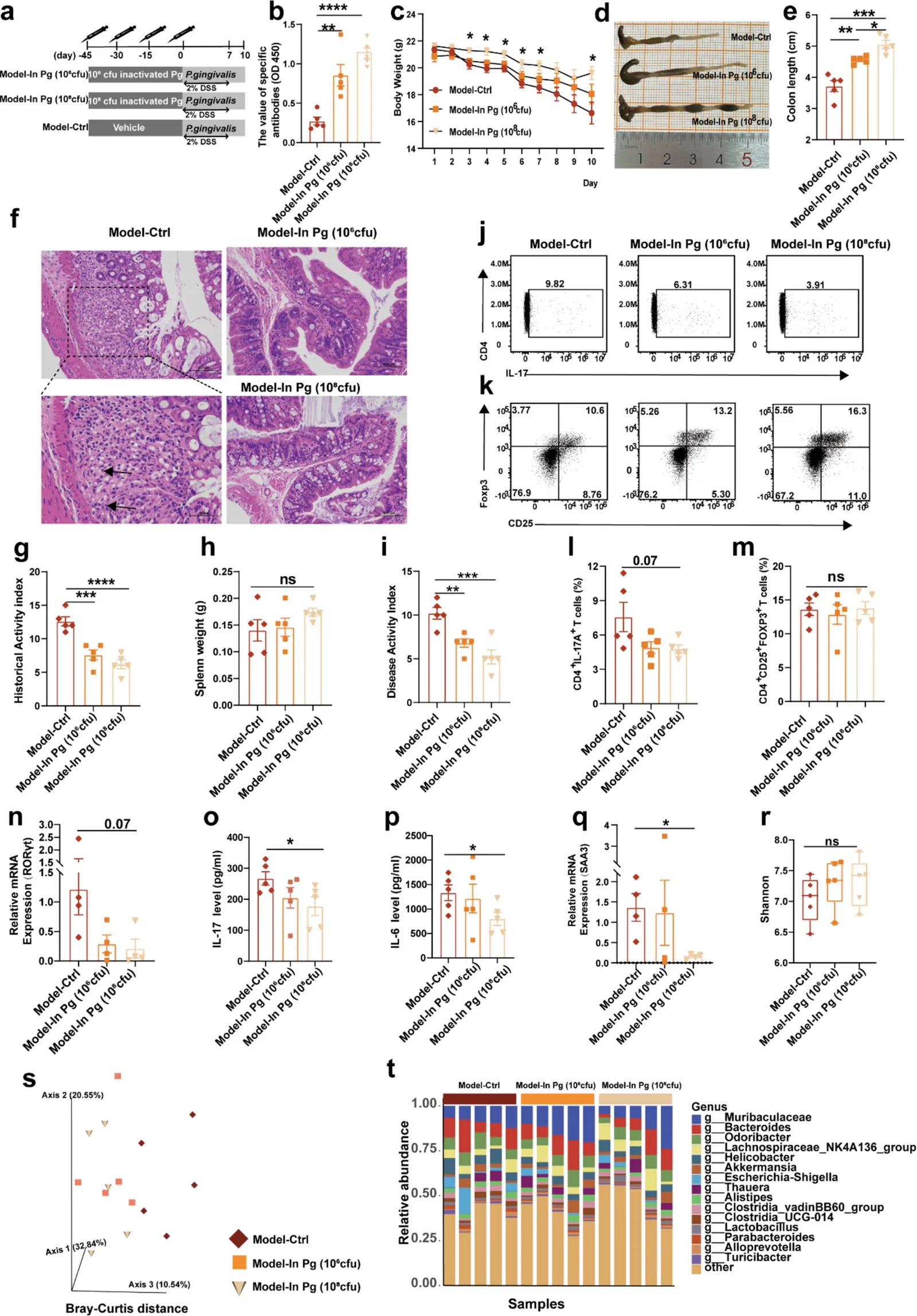
Immunization with inactivated Pg prevented Pg-aggravated intestinal inflammation. **a** The immunization experimental design. **b** The amount of specific antibody against Pg. **c** Body weight (*n* = 5 per group). **d**,** e** Representative macroscopic images of colons and statistical analysis of colon length. **f** H&E stained colon sections. **g** Histological scores of colons. Scale bar = 100 μm. The magnified inset shows the Model-Ctrl group (scale bar = 50 μm). Black arrows indicate neutrophil infiltration. **h** Spleen weight. **i** DAI (*n* = 5 per group). **j**–**m** The proportions of IL-17^+^ Th17 cells and Foxp3^+^ Treg cells and their ratio among total CD4.^+^ T cells within the spleens were detected by flow cytometry (*n* = 5 per group). **n** The relative mRNA expressions of RORγt (*n* = 4 per group). **o**–**p** Concentrations of IL-6 and IL-17 measured by ELISA (*n* = 5 per group). **q** The relative mRNA expressions of SAA3 (*n* = 4 per group). **r** The Shannon index. **s** PCoA based on Bray–Curtis distance of fecal microbiota. **t** The microbiota composition of feces at the genus level (*n* = 5 per group, two cages). The data were presented as the mean ± SEM and *p* values were assessed by one-way ANOVA with Tukey’s test. *p ≤ 0.05, **p ≤ 0.01, ***p ≤ 0.001

To further explore potential therapeutic strategies for Pg-aggravated intestinal inflammation, we performed subcutaneous immunization of the mice with 10^6^ CFU and 10^8^ CFU of inactivated Pg (Fig. 6a). We used ELISA to measure specific immunoglobulin G antibodies against Pg’s LPS (Fig. 6b). Compared to the Model-Ctrl group, both the Model-In Pg (10^6^ CFU) and Model-In Pg (10^8^ CFU) groups exhibited significant alleviation in colon length, weight loss, HAI, and DAI (Fig. 6c–i). Subcutaneous injection of inactivated Pg at 10^8^ CFU significantly attenuated the increase of Th17 cells (Fig. 6j–m), mRNA expression levels of RORγt (Fig. 6n), and Th17-related cytokine levels (IL-6, IL-17, and SAA3) (Fig. 6o–q). The mice’s fecal microbiota was characterized to investigate the effect of prophylactic treatment on Pg-infected mice. The α-diversity analysis with Shannon showed no differences among the groups (Fig. 6r). PCoA analysis revealed that although the Model-Ctrl group exhibited a distinct microbiota clustering pattern, differing from the Model-In Pg (10⁶ CFU) and Model-In Pg (10⁸ CFU) groups (Fig. 6s), there were no significant differences in the relative abundances of dominant genera among the three groups (Fig. 6t). These findings suggest that immunization with inactivated Pg could potentially mitigate Pg-induced the increase of Th17 cells and intestinal inflammation, while its impact on alleviating gut microbiota dysbiosis remains limited.

## Discussion

Emerging evidence has increasingly highlighted the oral–gut axis as a critical yet underappreciated pathway influencing intestinal homeostasis and disease [39]. Among various oral pathobionts, Pg has become a focal organism in oral–gut axis research, as growing evidence suggests that it can alter gut microbiota composition, disrupt intestinal barrier function, and activate mucosal immune responses [40].While ingested oral bacteria typically struggle to colonize the healthy intestine [41], gut inflammation can disrupt colonization resistance conferred by the resident microbiota, allowing ingested oral pathobionts to ectopically colonize the compromised intestine and influence disease progression [42]. Our results indicated that Pg may exacerbate DSS-induced intestinal inflammation, potentially by promoting Th17 cell expansion; notably, this inflammatory response was attenuated when the increase in Th17 cells was inhibited. Subsequently, we demonstrate that Th17-mediated inflammation may be caused by gut microbiota dysbiosis, as co-housing experiments showed that gut microbiota remodeling effectively inhibited Th17-driven inflammatory response. In contrast, the use of Th17 inhibitors did not have a significant effect on Pg-induced gut microbiota dysbiosis. Lastly, we observed a progressive decrease in the inflammatory and immune responses induced by Pg with the sequential knockout of individual gingipain genes, although statistical significance was reached only with the triple knockout strain (ΔKRAB). Taken together, these findings suggest that Pg may promote gut microbiota dysbiosis in a gingipain-dependent manner, which in turn facilitates the expansion of Th17 cells and the progression of intestinal inflammation.

In our study, the daily oral dose of Pg (10^9^ CFU) was chosen based on both experimental precedent and physiological relevance. This dosage has been widely adopted in previous investigations exploring the intestinal pathogenicity of oral bacteria in DSS-induced colitis models and other systemic inflammatory diseases. Importantly, the bacterial load is within the estimated physiological range, as individuals with periodontitis are known to swallow approximately 10^9^–10^13^ total bacteria per day through saliva [43, 44], among which Pg may account for 10^5^–10^11^ CFU [45, 46]. Moreover, evidence from Yamazaki et al. [12] demonstrated that, under comparable conditions, only Pg—but not other oral bacteria—induced significant intestinal inflammation and barrier disruption, underscoring the specificity of Pg’s pathogenic effects rather than a nonspecific consequence of bacterial gavage. Consistently, in our experiments, gingipain-deficient mutant strains of Pg, administered at the same bacterial dose, elicited markedly milder inflammatory responses compared with the wild-type strain. These findings collectively indicate that the observed inflammation is Pg-specific and gingipain-dependent, rather than a general response to bacterial exposure or DSS sensitization.

Interestingly, Pg alone did not induce intestinal inflammation or gut microbiota dysbiosis, supporting the hypothesis that oral microbiota may exist at low levels in a healthy gut, rendering them insufficient to exert pathogenic effects [47]. For instance, Zhi Wang et al. [11] reported higher levels of Pg in the intestines of CRC patients compared to healthy individuals. Consistently, our results revealed that the Pg levels in the colonic contents of the Pg-gavaged mice were not significantly different from those in the control mice. However, the DSS + Pg group exhibited significantly elevated Pg levels compared to both the PBS and DSS groups. Although Pg was detectable in the DSS + Pg group, this finding alone does not conclusively demonstrate whether Pg can persistently colonize or actively proliferate within the gut environment following the cessation of gavage administration. This limitation of our study highlights an important gap in understanding the dynamics of Pg colonization in the gastrointestinal tract. Without longitudinal tracking of Pg viability and abundance post-gavage, it remains unclear whether the pathogenic effects observed are a direct consequence of sustained bacterial presence or rather mediated by transient exposure triggering downstream immune or microbial dysbiosis responses. Future studies employing more sensitive and time-resolved detection methods, such as in vivo imaging or metagenomic sequencing, are needed to elucidate the temporal relationship between Pg colonization and its pathogenic role in gut inflammation. This challenge underscores the complexity of investigating bacterial pathogenicity in the intestinal environment. Nevertheless, we highlighted that the continuous delivery of Pg to the gut via saliva, even with its transient presence at a low abundance, may release derived molecules or disrupt the gut microbiota structure, potentially influencing disease progression. Supporting this, studies such as Kazuhisa Yamazaki et al. [12] have demonstrated that even the transient presence of Pg in the mouse gut can lead to microbiota dysbiosis, elevate blood endotoxin levels, and reduce intestinal tight junction protein levels.

Oral pathogens can contribute to the development of systemic diseases by triggering abnormal immune responses. Th17 cells play a crucial role in inflammation caused by commensal oral bacteria in both mice and humans [48, 49]. We found that inhibiting Th17 cells appeared to mitigate Pg-aggravated inflammation, suggesting that Pg may exacerbate intestinal inflammation in part by promoting Th17 cell expansion and associated pro-inflammatory responses. These findings are consistent with previous reports of Pg involvement in intestinal diseases, as well as studies on other oral bacteria. For instance, Pg has been shown to promote the progression of intestinal inflammation by inducing Th17-driven inflammatory response in both the spleen and gut [50, 51]. Similarly, Fusobacterium nucleatum and Parvimonas micra have been reported to trigger the differentiation of RORγt⁺ Th17 effector cells, thereby exacerbating colorectal cancer (CRC) [52, 53]. Given the significant Th17 cells observed in the spleens of mice, it is plausible that Pg-induced Th17 cells may undergo systemic migration, thereby contributing to the pathogenic mechanisms underlying Pg-associated systemic diseases. Yoshihiko Tanaka et al. [54] reported that intestinal-derived Th17 cells induced by Pg can migrate to the oral cavity, triggering periodontitis. Similarly, another study reported that Pg disrupted the Th17/Treg cell ratio in the colon of alcoholic fatty liver disease (ALD) mice, exacerbating systemic immune dysregulation and inflammatory responses through the “oral-gut-liver” axis [40]. Moreover, immunization with heat-inactivated Pg induced specific antibodies that modulated the host immune response, notably by limiting Pg-driven Th17 activation and alleviating subsequent intestinal inflammation. Previous studies have also reported that Pg-specific antibodies can prevent or alleviate other Pg-mediated systemic diseases. For example, Rémy Burcelin and colleagues [8] demonstrated that specific antibodies generated in mice immunized with inactivated Pg protected the animals from metabolic disorders induced by periodontitis. These studies highlight the complex interactions between oral pathogens and systemic immune responses. Understanding these mechanisms could provide novel insights into therapeutic strategies for preventing and treating systemic diseases influenced by oral pathogens.

Concomitantly with Th17 dysregulation, we observed that Pg significantly exacerbated DSS-induced gut microbiota dysbiosis in a gingipain-dependent manner, characterized by an increased proportion of Bacteroidetes and Escherichia-Shigella, alongside a reduction in Muribaculaceae. Related studies have reported similar findings. For instance, oral administration of Pg in mice led to an increased abundance of Bacteroides in the ileum [12], which is considered a marker of ulcerative colitis [55]. Similarly, oral Pg exposure has been shown to increase the levels of Escherichia-Shigella and decrease Muribaculaceae in mice with alcoholic fatty liver disease [40]. Our co-housing experiment demonstrated that coprophagy, and the resulting microbiome remodeling, conferred protection against inflammation in Pg-treated mice when they cohabitated with healthy control mice. Combined with the observation that Th17 inhibition did not alleviate Pg-mediated gut microbiota dysbiosis, these findings further support the notion that Pg-induced gut microbiota dysbiosis may trigger aberrant immune cell responses, rather than the reverse. This mediatory role of gut microbiota dysbiosis aligns with the findings of Yoshihiko Tanaka et al. [54], which revealed that Pg did not induce the increase of Th17 cells in germ-free mice. Several studies have reported specific gut microbes and their mechanisms that regulate Th17-mediated immune responses, including the enzyme cgr2 secreted by Eggerthella lenta [56] and butyric acid produced by Clostridium [57]. These results suggest intricate, bidirectional interactions between cellular immune regulation and the host-associated microbiome. However, the precise mechanisms by which Pg-induced gut microbiota dysbiosis contributes to immune dysregulation warrant further investigation to pinpoint the key causal microbes and metabolites.

Gingipains, cysteine proteases secreted by Pg that specifically cleave peptide bonds following lysine or arginine residues, serve as primary virulence factors implicated in various Pg-associated systemic diseases. Using gingipain knockout strains, we found that gingipains may contribute to gut microbiota dysbiosis and potentially promote Th17 cell expansion. In line with this observation, Jan Potempa et al. [58] reported that Pg can influence the progression of aspiration pneumonia in a gingipain-dependent manner. Likewise, Gabriel Nussbaum et al. [14] detected gingipains in pancreatic cancer tissues, with evidence suggesting a possible role in tumor progression in mice. Similar to Pg gingipains, other pathobionts have been reported to exert virulence through their proteases. For instance, the metalloprotease GelE produced by the commensal Enterococcus faecalis strain can degrade the extracellular domain of the adhesion protein E-cadherin, leading to the disruption of epithelial barrier integrity and the onset of intestinal inflammation in susceptible mouse models [59]. Additionally, proteases from certain Bacteroides species have been implicated in the pathogenesis of UC. For example, broad-spectrum protease inhibitors have been shown to rescue Bacteroides vulgatus-induced epithelial barrier dysfunction in vitro and to attenuate B. vulgatus-driven colitis in IL-10–deficient mice, suggesting that bacterial proteolytic activity may contribute to UC pathogenesis [60]. However, the exact mechanism by which gingipain impacts the gut microbiota remains elusive, highlighting a limitation of this study. Gingipains can proteolytically cleave host proteins and facilitate the uptake of amino acids, thereby altering the host microenvironment [61]. Noah W. Palm et al. [62] demonstrated that catalytically active gingipain K from Pg activates CD97/ADGRE5—a member of the adhesion G protein–coupled receptor (GPCR) family—through cleavage at the K290 site. Given that GPCRs are common targets of gut microbiota–derived metabolites, this finding raises the possibility that gingipains may interfere with host–microbiota interactions by disrupting metabolite–GPCR signaling. Together, gingipains may induce dysbiosis of the host-microbiota interaction through multiple mechanisms, necessitating further research to elucidate the specific contributions of each mechanism.

In conclusion, this study expands our understanding of the mechanisms through which Pg aggravates intestinal inflammation, providing important evidence for the role of human oral microbial translocation in driving gastrointestinal diseases and paving the way for the development of effective interventions targeting the contributions of oral microbiota to gut and systemic diseases.

## Supplementary Information

Supplementary Material 1.

## Data Availability

The 16S rRNA amplicon sequencing data have been deposited in the BioProject database at CNCB under accession number PRJCA026233. The authors state that all additional data supporting the findings of this study are included within the article and its Supplementary Information file or can be obtained from the corresponding author upon request.

## Abbreviations

Pg: *Porphyromonas gingivalis*
IBD: Inflammatory bowel disease
CRC: Colorectal cancer
DAI: Disease activity index
HAI: Histological activity index
ZO-1: Zonula occludens-1
MUC-2: Mucin-2
SAA: Serum amyloid A proteins
Kgp: Lysine-specific gingipain
RgpA: Arginine-specific gingipain A
RgpB: Arginine-specific gingipain B
Th17 cells: T helper 17 cells
Treg cells: Regulatory T cells
DSS: Dextran sodium sulfate
