# Correction: *Porphyromonas gingivalis* induces intestinal inflammation through gingipain-dependent gut microbiome dysbiosis

**DOI:** 10.1186/s40168-026-02532-4

**Published:** 2026-09-04

**Authors:** Ming Li, Jiyun Cui, Ru Qu, Rulong Liu, Yifan Sun, Ping Li, Juan Liu, Adrian Low, Xiaochang Huang, Fei Gan, Zhenjiang Zech Xu

**Affiliations:** 1 Shenzhen Hospital, Southern Medical University, Shenzhen, Guangdong China; 2 College of Food and Bioengineering, Bengbu University, Bengbu, China; 3 State Key Laboratory of Food Science and Resources, No. 235 Nanjing East Road, Nanchang, Jiangxi 330047 China; 4 Hubei Key Laboratory of Cell Homeostasis, College of Life Science, TaiKang Center for Life and Medical Sciences, Wuhan University, Wuhan, China; 5 Institute of Biological Resources, Jiangxi Academy of Sciences, Nanchang, 330096 China; 6 Department of Medicine, Yong Loo Lin School of Medicine, National University of Singapore, MD6 Centre for Translational Medicine, 14 Medical Drive, Singapore, 117599 Singapore

**Correction: Microbiome 14, 148 (2026)**

**https://doi.org/10.1186/s40168-026-02389-7**

Following publication of the original article [1], the author reported that during figure assembly, the staining image for the DSS group in Fig. 3f was inadvertently replaced with the staining image from the Co-PBS group in Fig. 5f.

The incorrect Figure 3F was

**Figure:**
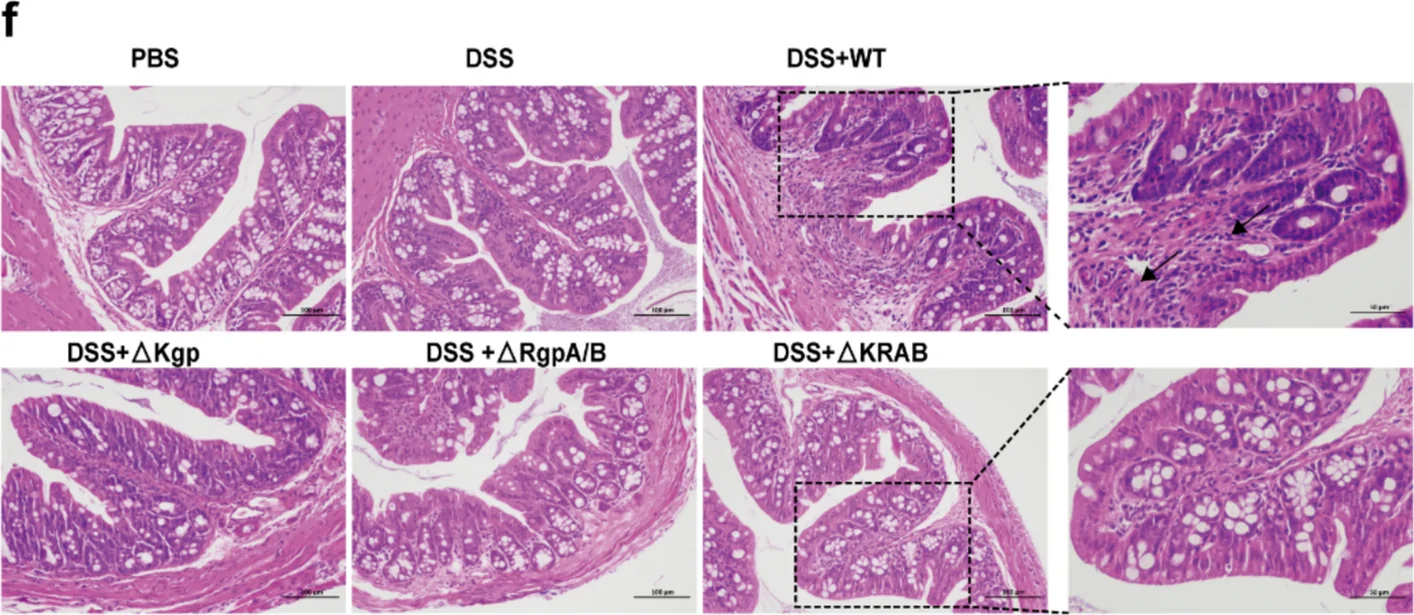

The correct Figure 3F is

**Figure:**
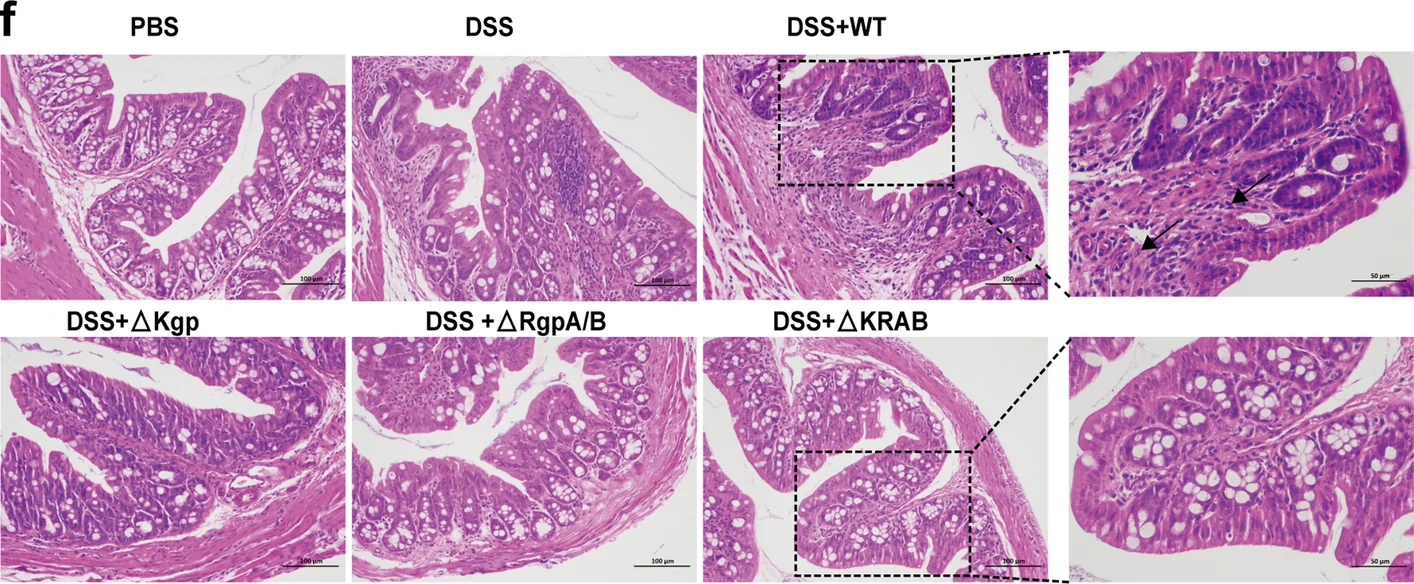

The original article has been updated.
