# Supplementary material for: Porphyromonas gingivalis induces intestinal inflammation through gingipain-dependent gut microbiome dysbiosis
Source: Microbiome. 2026 Apr 2;14:148. doi: 10.1186/s40168-026-02389-7 (PMC13181988; doi:10.1186/s40168-026-02389-7)
Supplement: Supplementary file 2 — Supplementary Material 1. [file 40168_2026_2389_MOESM1_ESM.docx]

Supplementary Materials for

**Periodontal Pathogen Induces Gingipain-Dependent Gut Microbiome Dysbiosis in Exacerbating Intestinal Inflammation**

Ming Li *et al.*

*Corresponding author. Email: zhenjiangxu@ncu.edu.cn

**This PDF file includes:**

Figs. S1 to S15

Tables S1 to S3


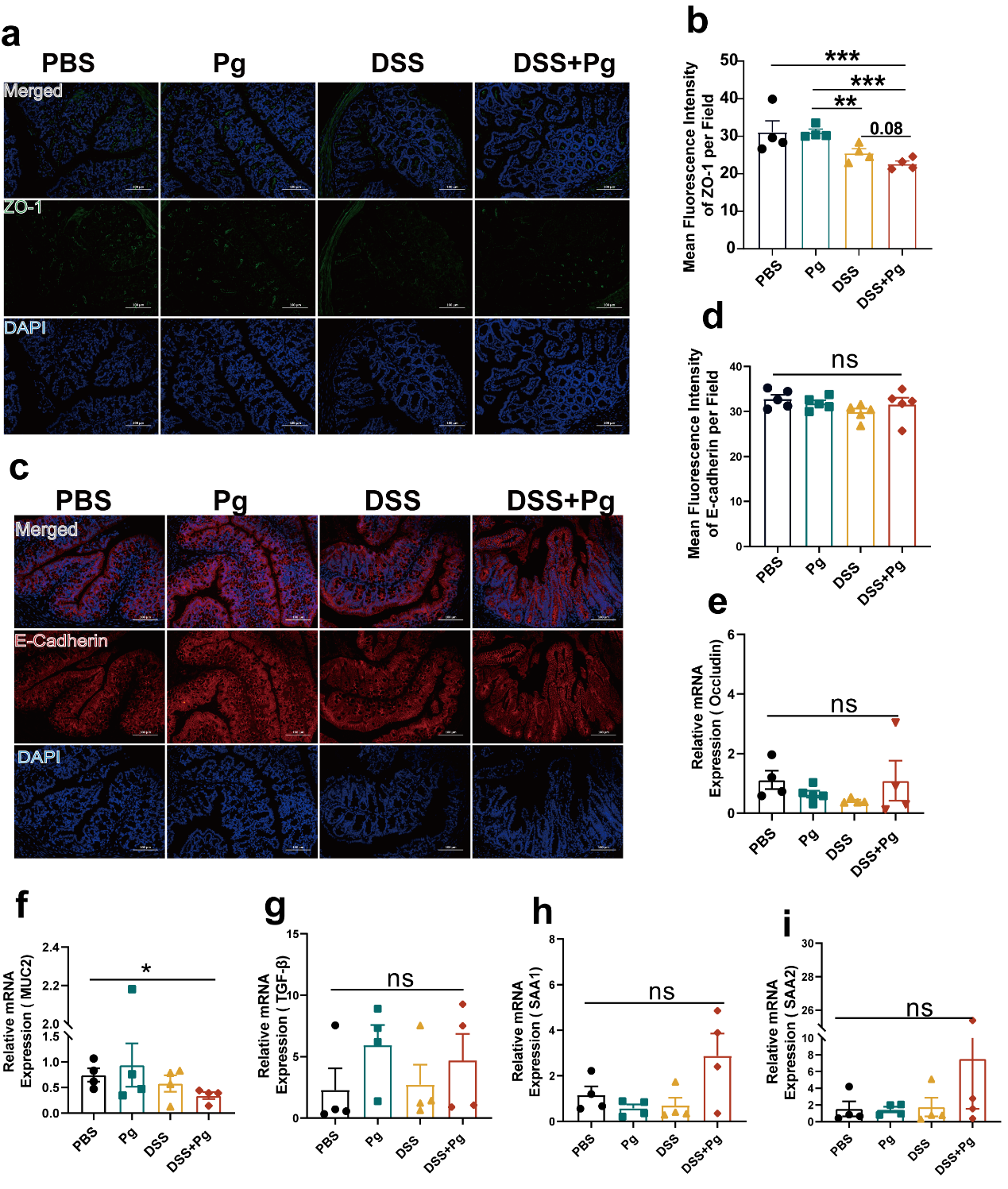


**Supplementary Fig. 1.** **a** Representative immunofluorescence images and quantification of ZO-1 in colon sections. ZO-1 was stained in green and nuclei are counterstained with DAPI (blue). **b** The expression levels of ZO-1 were quantified as Mean Fluorescence Intensity (MFI) per field (n=4 per group). **c** Representative immunofluorescence images and quantification of E-cadherin in colon sections. E-cadherin is stained in red and nuclei are counterstained with DAPI (blue). Scale bar=100μm.  **d** The expression levels of E-cadherin were quantified as Mean Fluorescence Intensity (MFI) per field (n=5 per group). **e-i** The relative mRNA expressions of Occludin (**d**), Muc2 (**e**), TGF-β (**f**), SAA1 (**g**), and SAA2 (**h**) in colon tissues in the Pg treatment experiment (n=4 per group). The data were presented as the mean ± SEM and evaluated by one-way ANOVA with Tukey’s test. **p* ≤ 0.05, ***p* ≤ 0.01, ****p* ≤ 0.001.


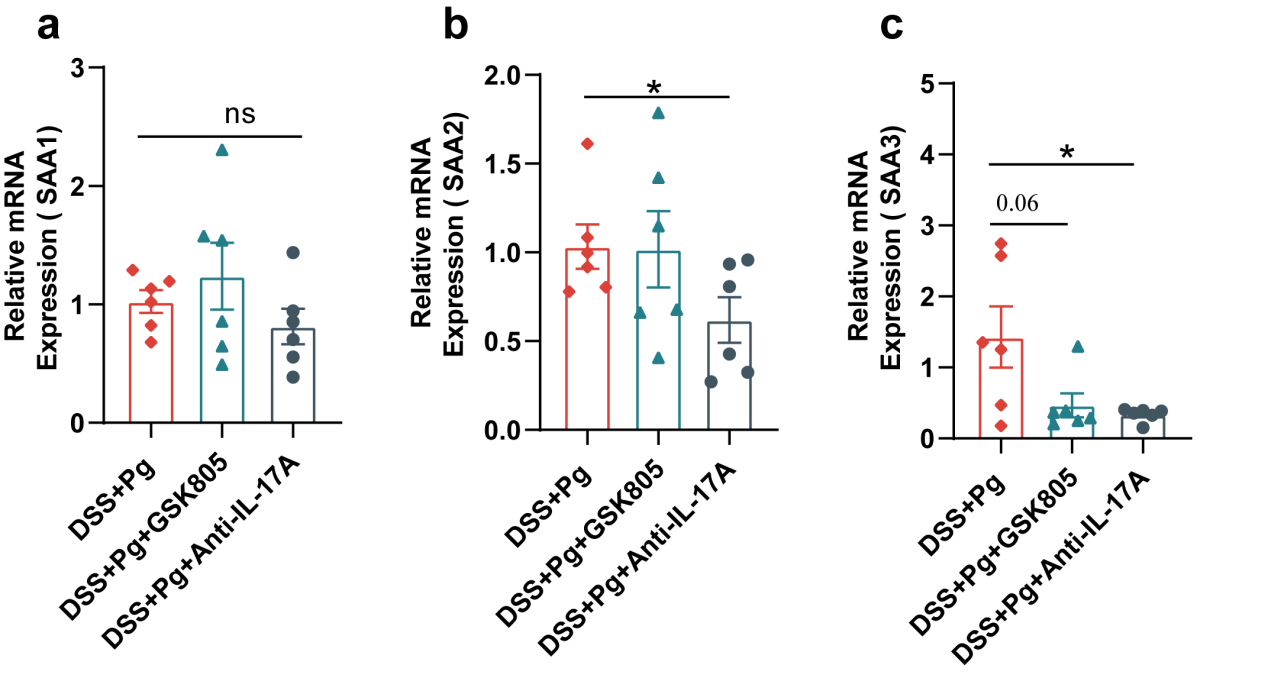


**Supplementary Fig. 2**. a-c The relative mRNA expressions of SAA1 (a), SAA2 (b), and SAA3 (c) in colon tissues from the Th17 inhibition experiment. The data were presented as the mean ± SEM and evaluated by one-way ANOVA with Tukey’s test. n=6 per group. **p* ≤ 0.05, ***p* ≤ 0.01, ****p* ≤ 0.001.


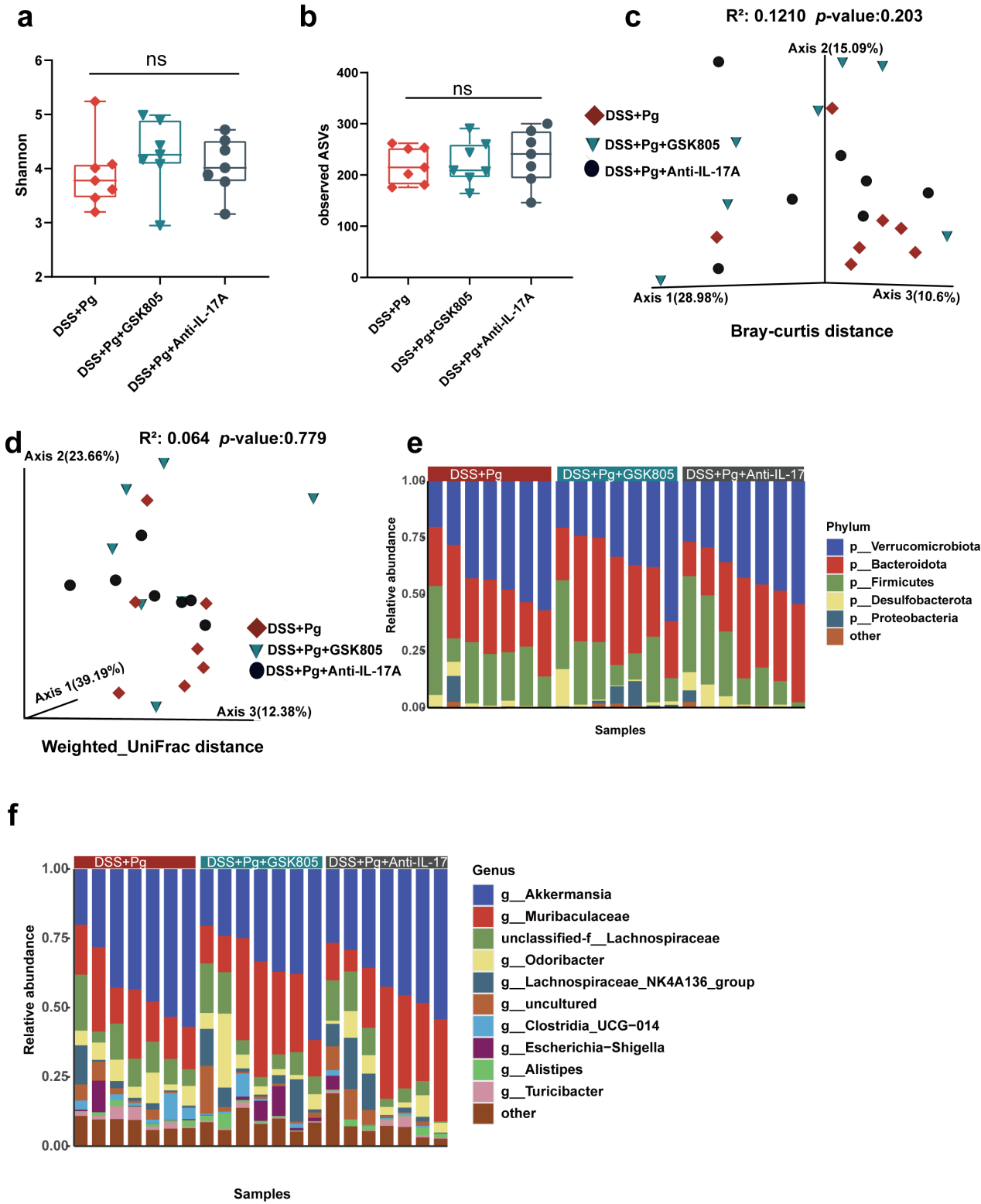


**Supplementary Fig. 3**. The impact of Th17 inhibition on the gut microbiota of mice. **a-b** Alpha diversity analysis of the gut microbiota from the Th17 inhibition experiment measured with the Shannon index and observed ASVs. **c-d** Bray-Curtis and Weighted UniFrac distances based PCoA of the gut microbiota from the Th17 inhibition experiment. **e-f** The microbiota composition at the phylum and genus levels. n=7 per group, two cages. **p* ≤ 0.05, ***p* ≤ 0.01, ****p* ≤ 0.001.


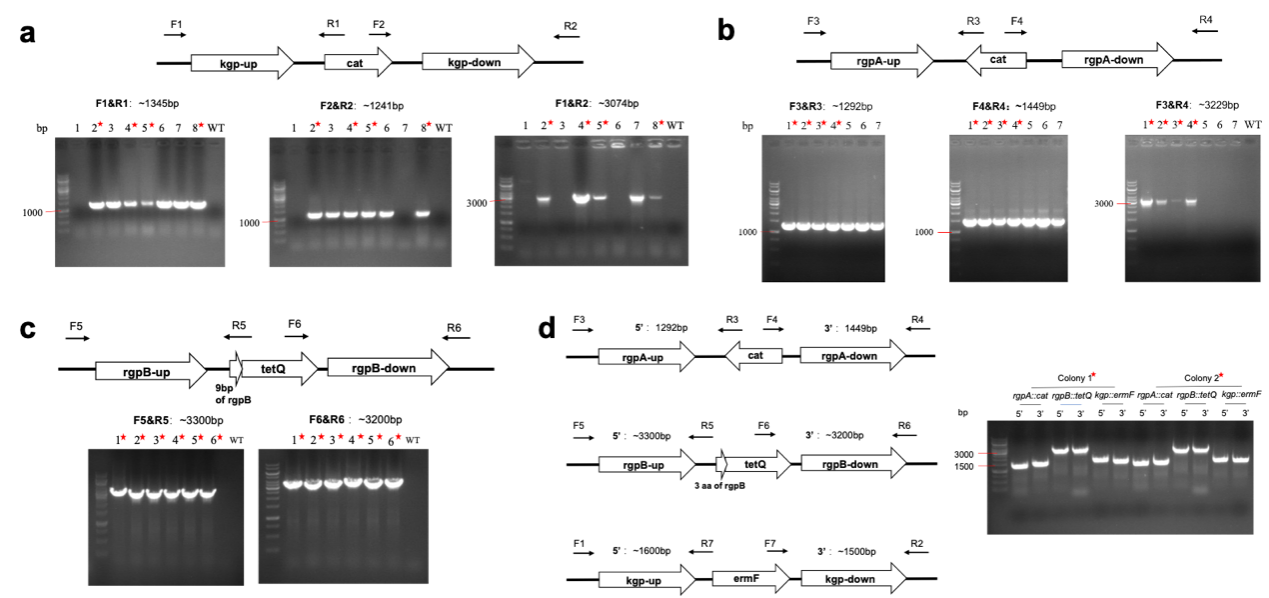


**Supplementary Fig. 4**. PCR confirmation of gingipain mutants. **a** Validation of single knockout mutant ▲Kgp (Pg*.*W83 △*kgp::cat*). **b** Validation of single knockout mutant ▲RgpA (Pg*.*W83 △*rgpA::cat*). **c** Validation of double knockout mutant ▲RgA/B (Pg*.*W83 △*rgpA::cat* △*rgpB::tetQ*). The gene *rgpB* was knocked out in ▲the RgpA mutant*.* **d** Validation of the triple knockout mutant ▲KRAB (Pg. W83 △*rgpA::cat* △*rgpB::tetQ* △*kgp::ermF*)*.* The gene *kgp* was knocked out in the ▲RgA/B mutant. The red star indicated the correct colony.


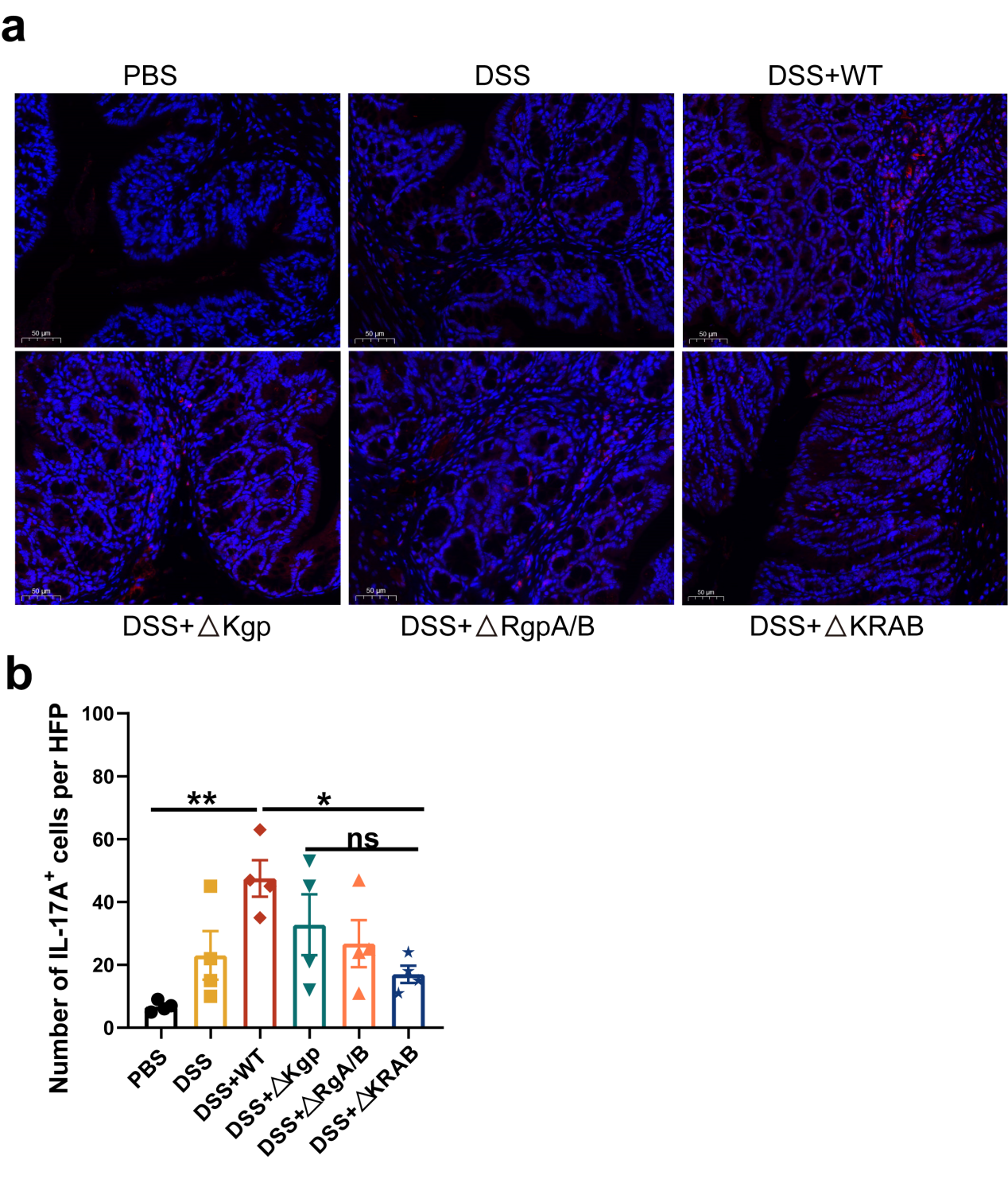


**Supplementary Fig. 5 a** Representative immunofluorescence images of colonic sections. IL-17A is labeled in red, and nuclei are counterstained with DAPI (blue). Scale bar = 50μm; **b** Quantification of IL-17A^+^ cells. Cell numbers were counted across the entire scanned area. Data are expressed as the number of IL-17A^+^ cells per high-power field (HPF, at 40 magnification) (mean ± SEM, n = 4 per group).


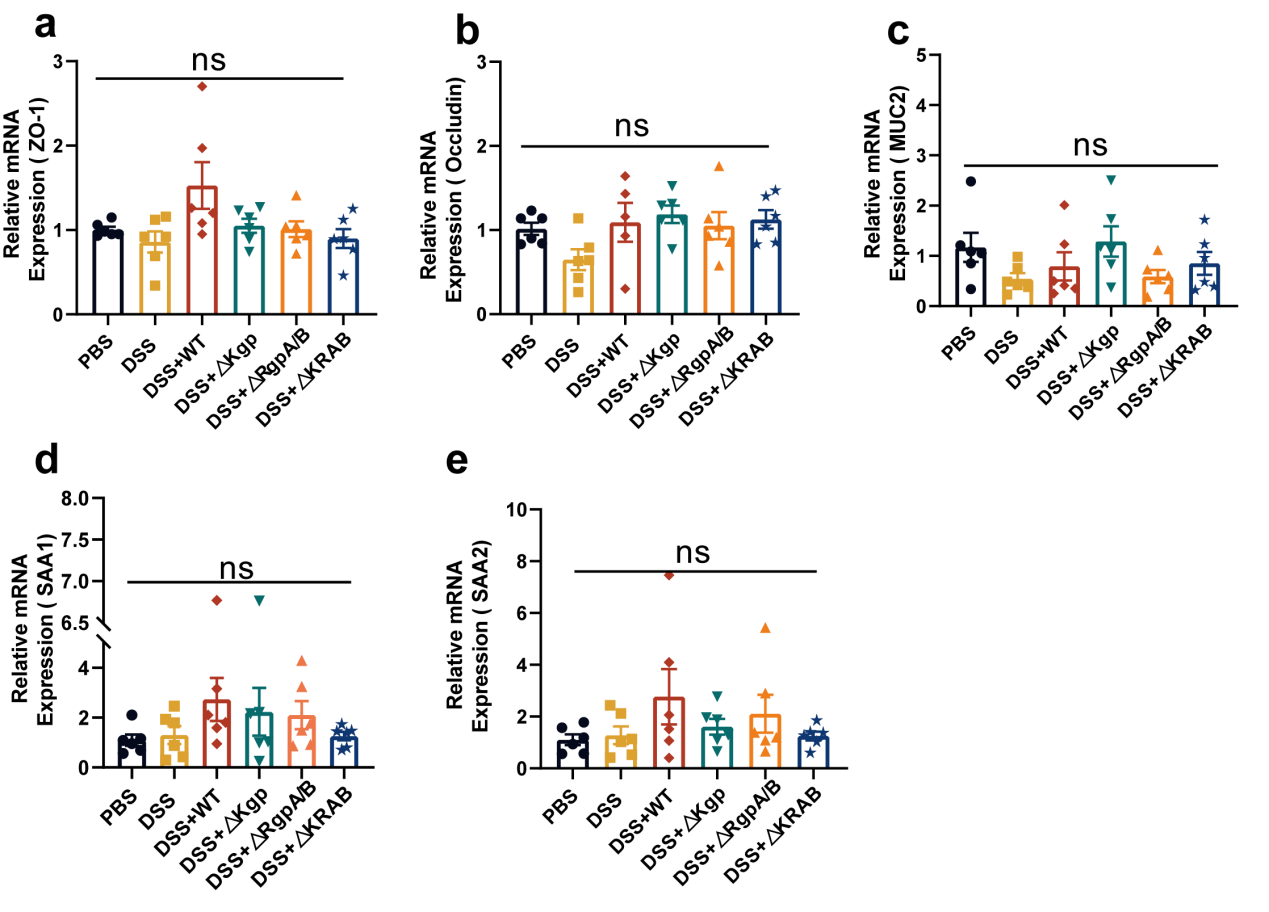


**Supplementary Fig. 6**. **a-e** The relative mRNA expressions of ZO-1 (**a**), Occludin (**b**), Muc2 (**c**), SAA1 (**d**), and SAA2 (**e**) in colon tissues from the gingipain-knockout strains experiment. The data were presented as the mean ± SEM and evaluated by one-way ANOVA with Tukey’s test. n=6 per group. **p* ≤ 0.05, ***p* ≤ 0.01, ****p* ≤ 0.001.


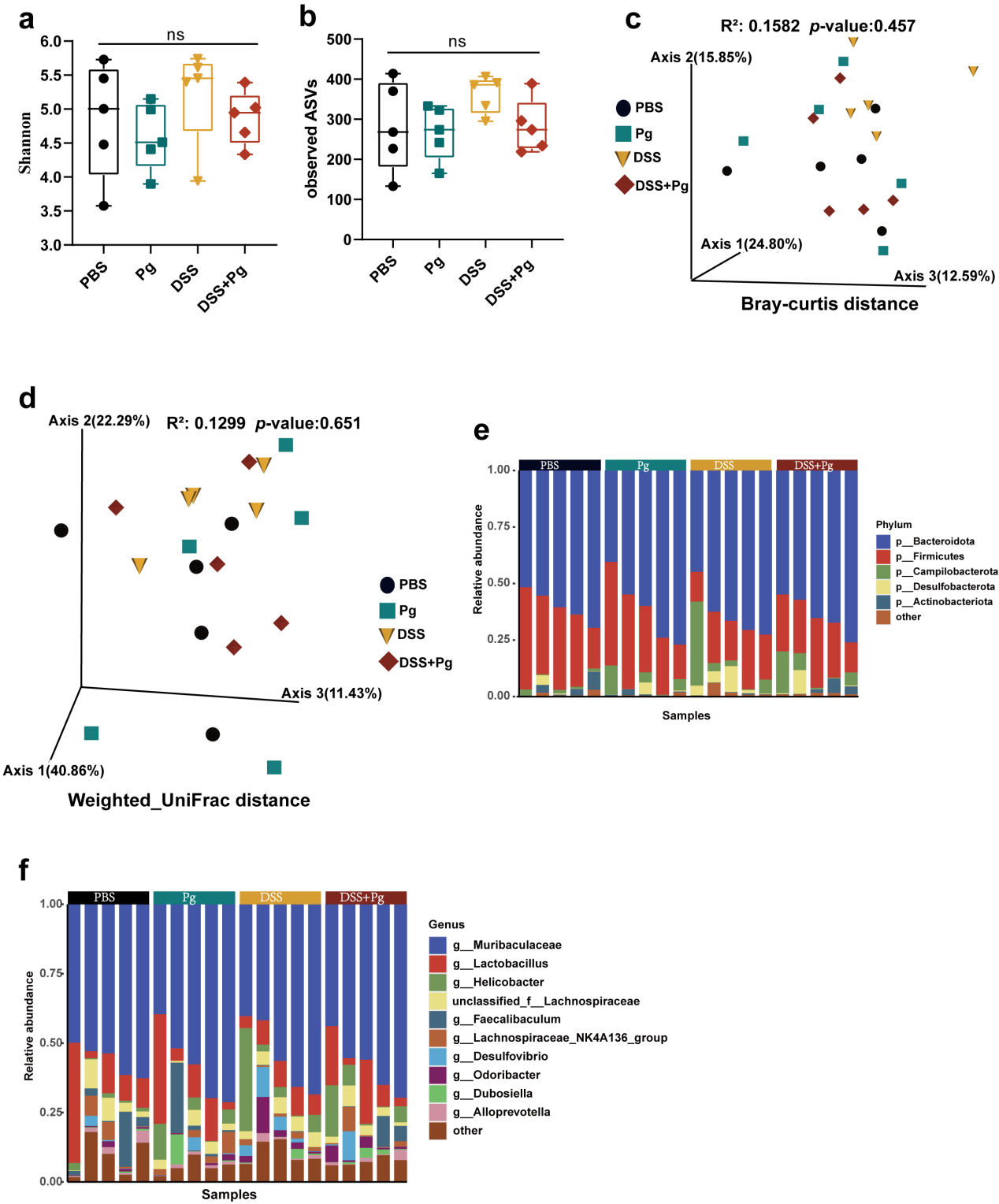


**Supplementary Fig. 7.** The background microbiota of mice before treatment. **a-b** Alpha diversity analysis of the gut microbiota from the Pg treatment experiment measured with Shannon index and observed ASVs. **c-d** Bray-Curtis and Weighted UniFrac distances based PCoA of the gut microbiota from the Pg treatment experiment. **e-f** The microbiota composition at the phylum and genus levels. n=5 per group, two cages.


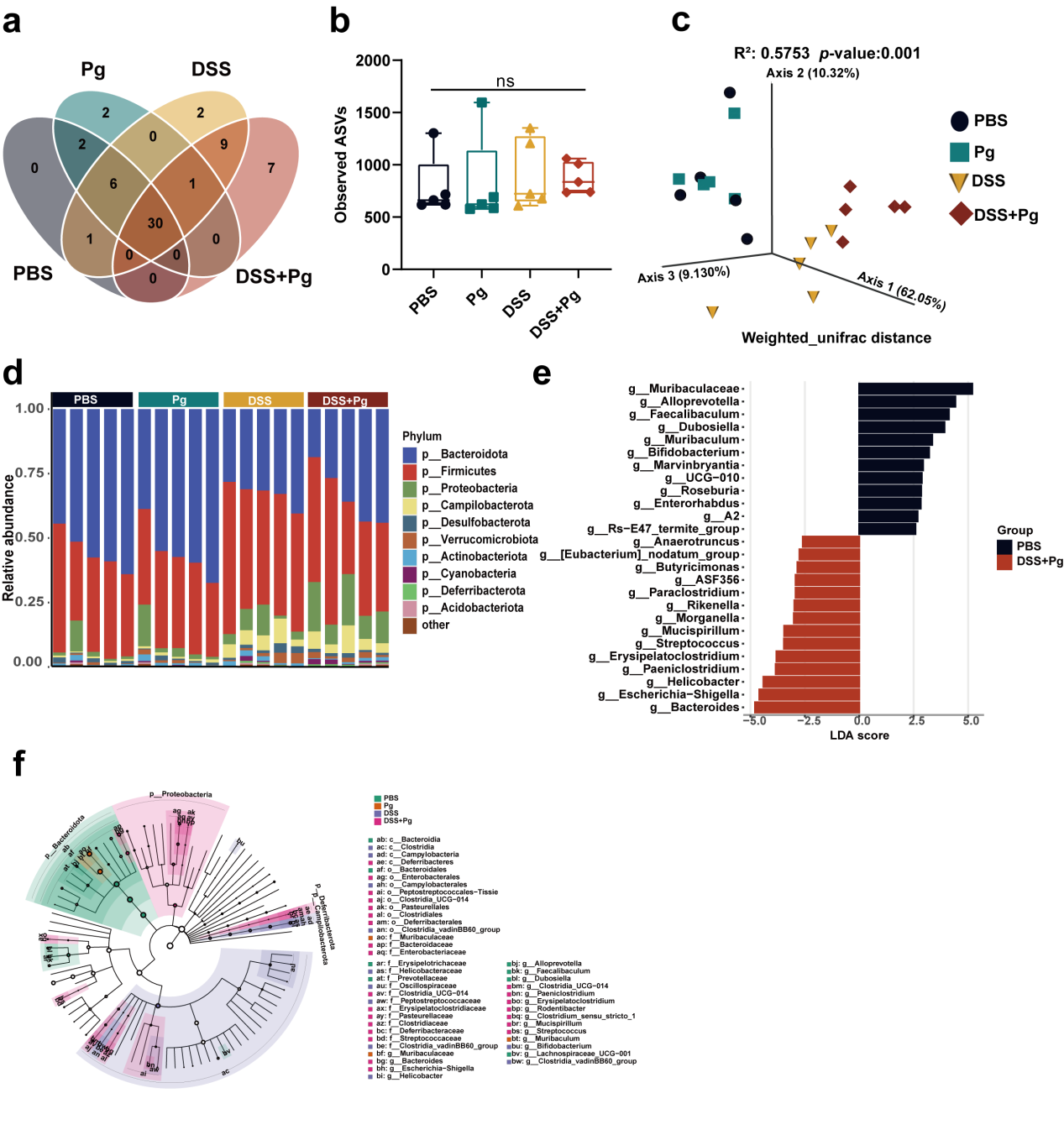


**Supplementary Fig. 8**. **a** The number of common or unique genera among different groups in the Venn diagram from the Pg treatment experiment. **b** Alpha diversity analysis of gut microbiota from the Pg treatment experiment measured with observed ASVs. **c** Weighted_UniFrac distance based PCoA of gut microbiota from the Pg treatment experiment. **d** Bacterial taxa of differential abundance among groups with the LDA scores derived from LEfSe analysis (LDA > 2.0) in the Pg treatment experiment. **f** LEfSe taxonomic cladogram illustrating taxa of differential abundances between microbiome communities.


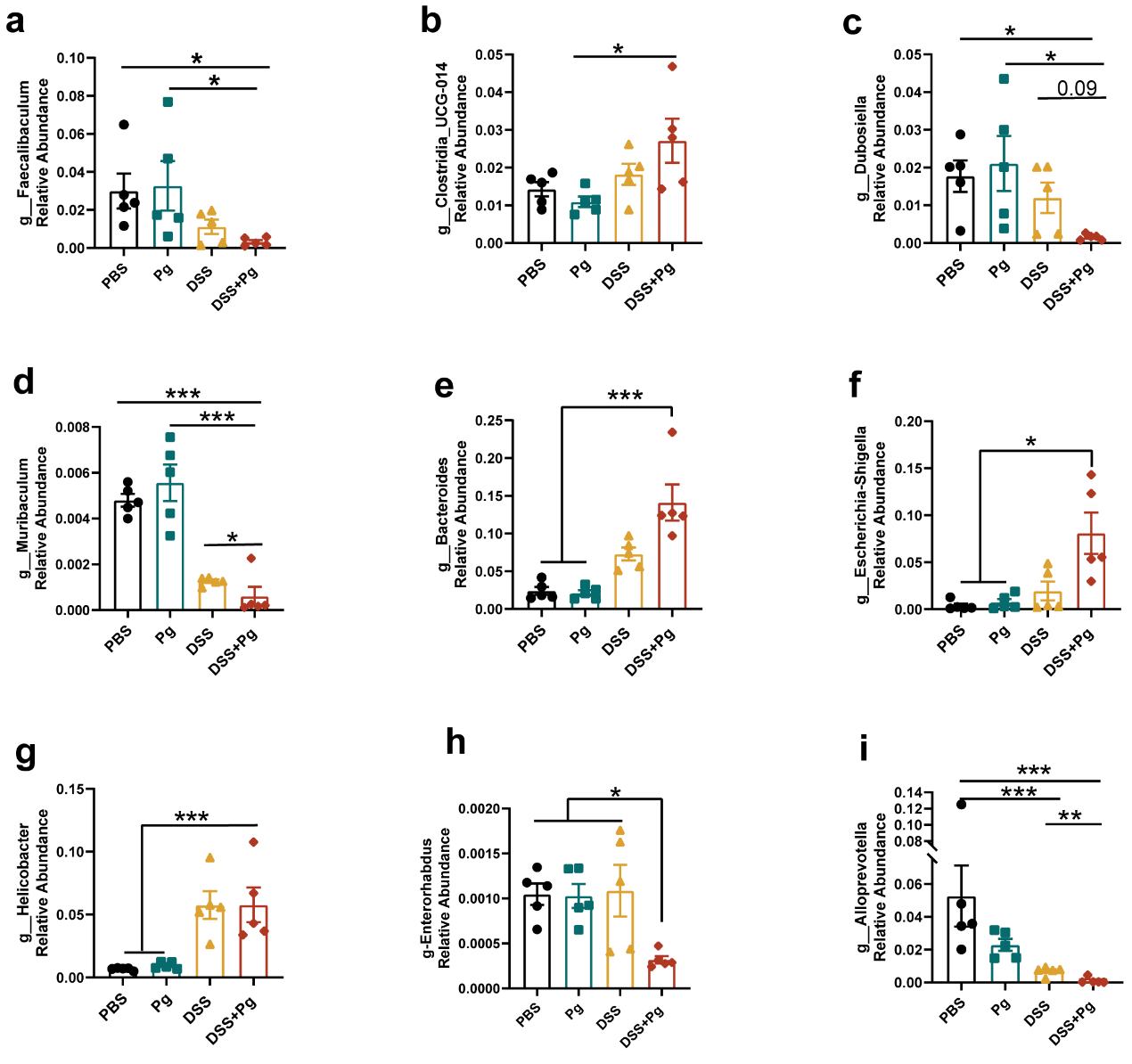


**Supplementary Fig. 9** **a-i** The box plots indicate the relative abundances of each bacterial group at the genus level in the Pg treatment experiment analyzed using Maaslin2 (*Faecalibaculum*, *Clostridia_UCG-014*, *Dubosiella*, *Muribaculum*, *Bacteroides*, *Escherichia-Shigella*, *Helicobacter*, *Enterorhabdus*, and *Alloprevotella*). n=5 per group, two cages. **p* ≤ 0.05, ***p* ≤ 0.01, ****p* ≤ 0.001.


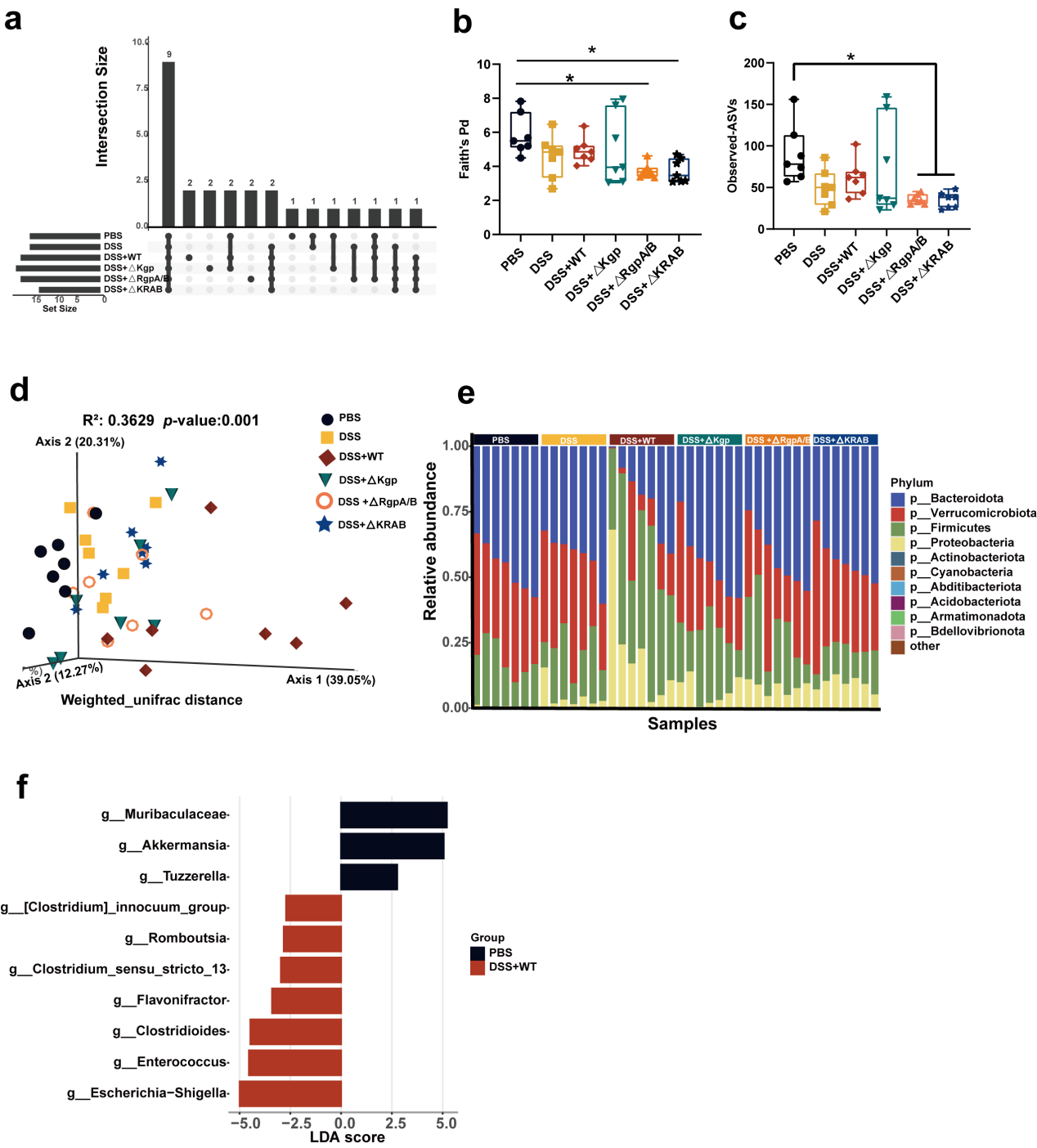


**Supplementary Fig. 10**. **a** The number of common or unique genera among different groups in the Venn diagram in the gingipain-knockout strains experiment. **b c** Alpha diversity analysis of gut microbiota measured with Faith’s PD and Observed ASVs in the gingipain-knockout strains experiment. **d** Weighted UniFrac distance-based PCoA of gut microbiota in the gingipain-knockout strains experiment. **e** The microbiota composition at the phylum level. **f** Bacterial taxa of differential abundance among groups with the LDA scores derived from LEfSe analysis (LDA > 2.0) in the gingipain-knockout strains experiment.


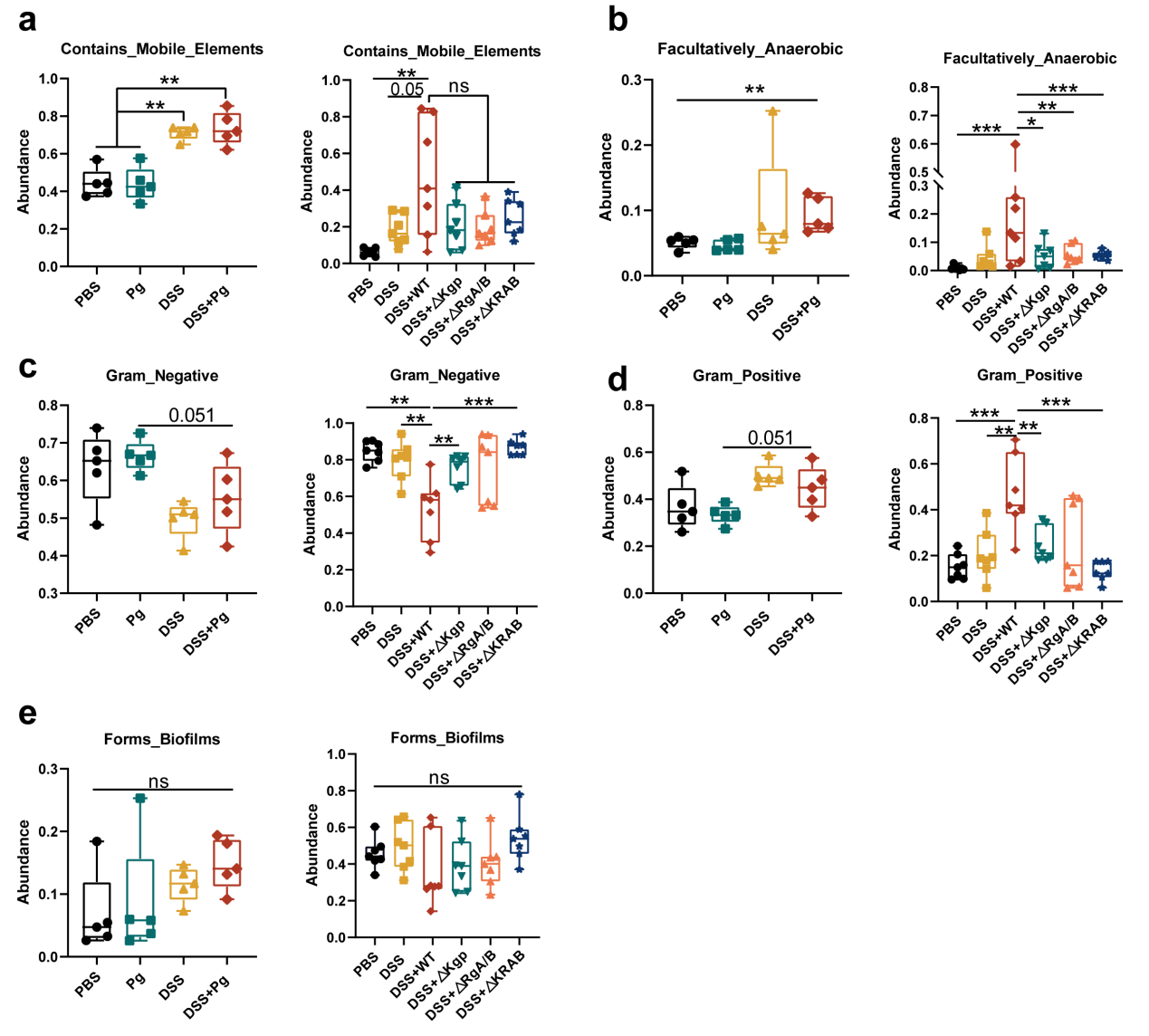


**Supplementary Fig. 11.** Gut microbiota phenotype prediction based on Bugbase was conducted for the Pg treatment experiment (left) and gingipain-knockout strains experiment (right). **a** Contains mobile element. **b** Facultatively anaerobic. **c** Gram negative. **d** Gram positive. **e** Forms_biofilms. **p* < 0.05; * * *p*< 0.01 and * ***p* < 0.001


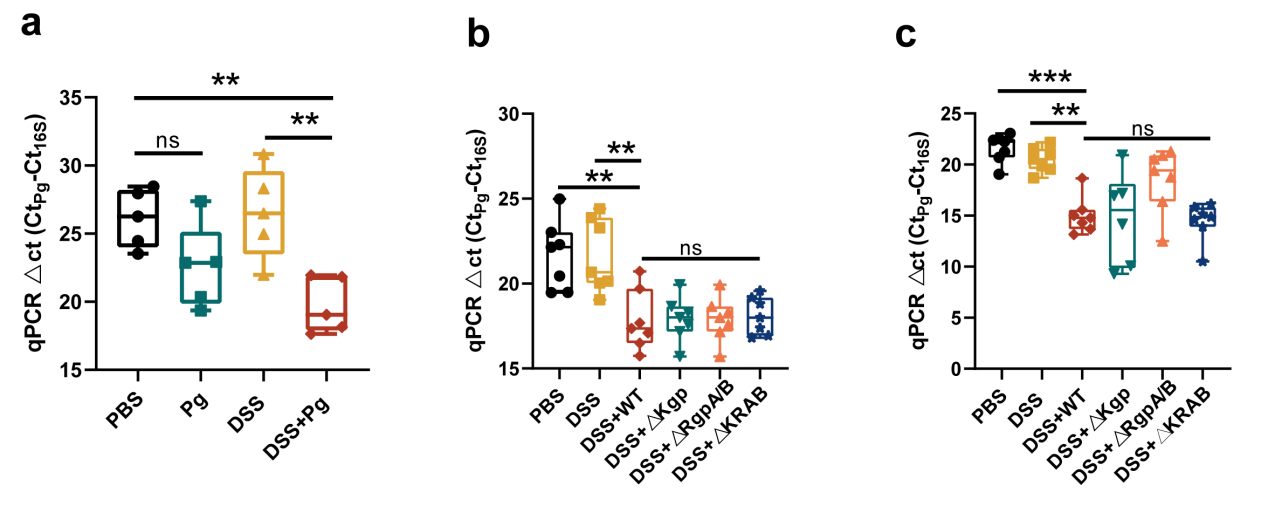


**Supplementary Fig. 12**. The relative levels of Pg in colonic tissues and colonic contents. **a** The Pg levels in the colonic contents of mice from the Pg treatment experiment (n=5 per group, two cages). **b** The Pg levels in colonic contents from mice in the gingipain-deficient Pg animal experiment; **c** The Pg levels in the colonic tissues from mice in the gingipain-deficient Pg animal experiment. The ΔCT is calculated by subtracting the CT value of the 16S universal primer from the CT value of the Pg-specific primer. A lower ΔCT value indicates a higher relative abundance of Pg(n=7 per group, two cages). **p* ≤ 0.05, ***p* ≤ 0.01, ****p* ≤ 0.001.


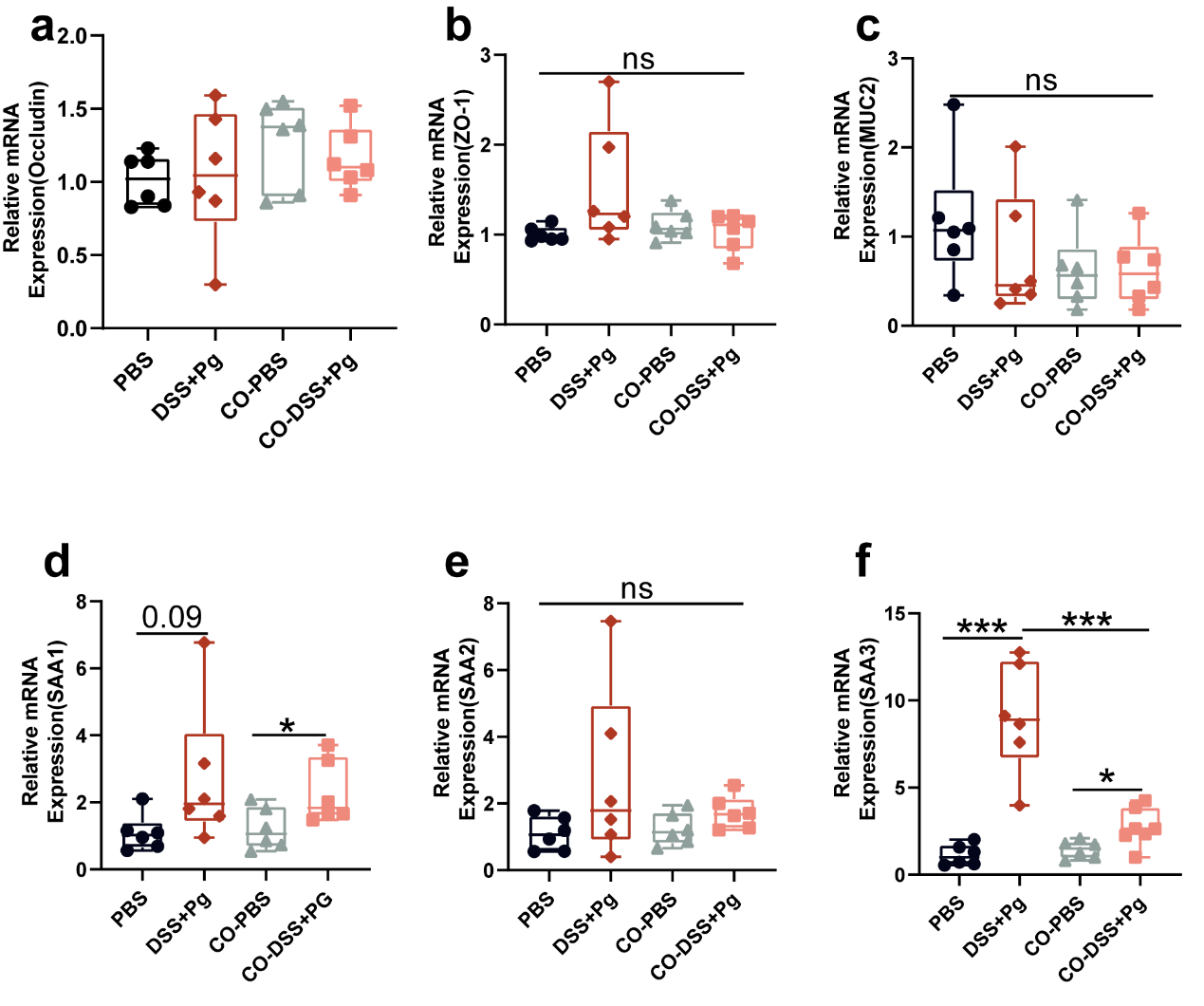


**Supplementary Fig. 13. a-f** The relative mRNA expressions of Occludin (**a**), ZO-1(**b**), MUC2 (**c**), SAA1 (**d**), SAA2 (**e**), SAA3 (**f**) in colon tissues from the co-housing experiment. The data were presented as the mean ± SEM and evaluated by one-way ANOVA with Tukey’s test. n=6 per group. **p* ≤ 0.05, ***p* ≤ 0.01, ****p* ≤ 0.001


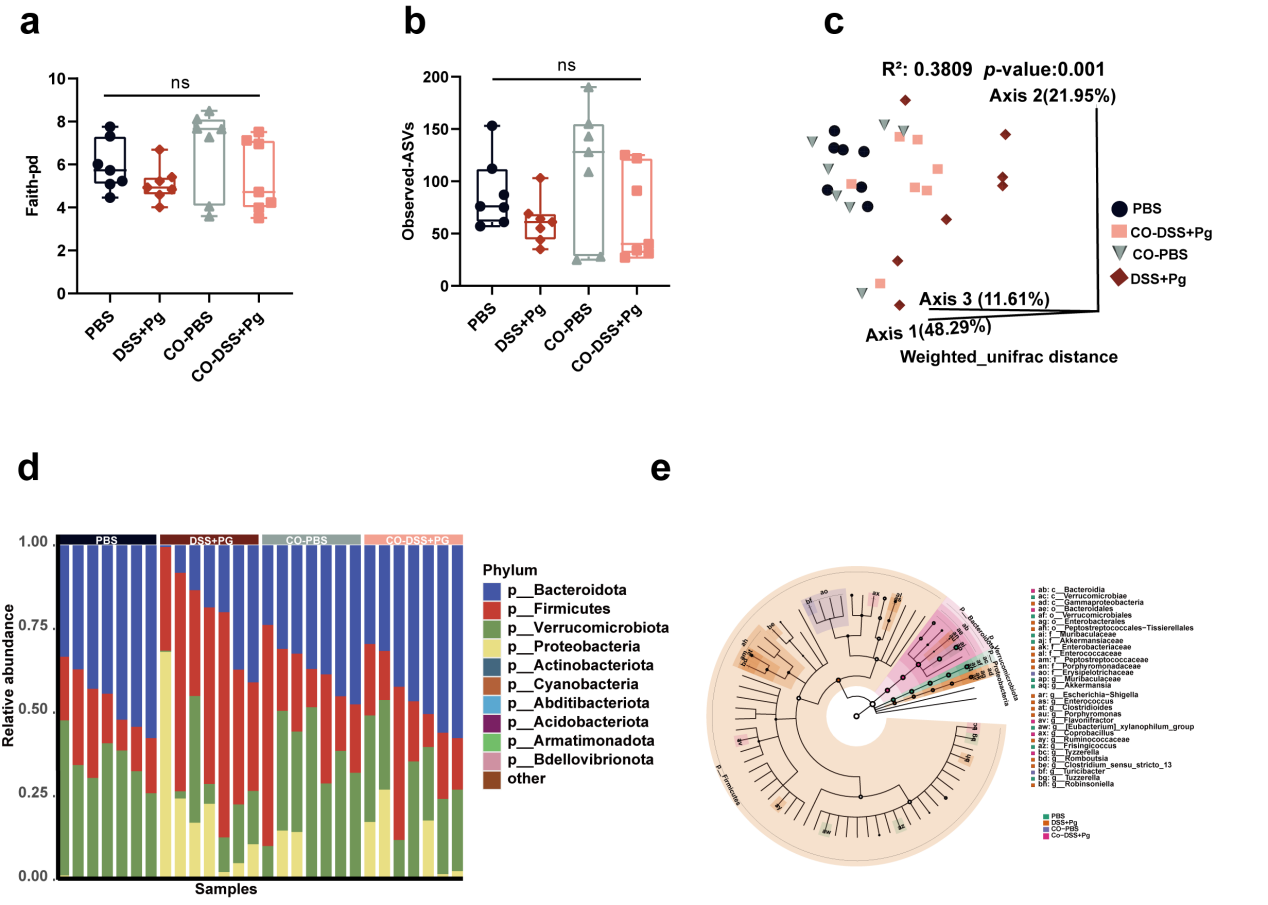


**Supplementary Fig. 14**. **a** Alpha diversity analysis of gut microbiota in the co-housing experiment measured with Faith’s PD and Observed ASVs. **c** Weighted UniFrac distance based PCoA of gut microbiota in the co-housing experiment. **d** The microbiota composition of feces at the phylum level. **e** LEfSe taxonomic cladogram illustrating taxa of differential abundances between microbiome communities.


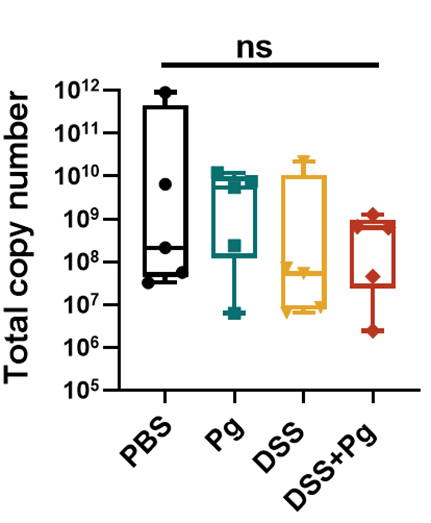


**Supplementary Fig. 15** The total bacterial copy number in the Pg treatment experiment. n=5 per group, two cages.

**Table S1 The plasmids used in this study.**

| **plasmid** | **antibiotic resistance** |
| --- | --- |
| pUC19 | Amp |
| pKN-kgp-cat | Cm |
| pKN-rgpA-cat | Cm |
| pUC19-rgpB-tetQ | Amp，Tet |
| pUC19-kgp-ermF | Amp，Em |

**Table S2 The primers used for the construction of knockout mutants.**

| **Primer** | **Sequence (5'→3')** |
| --- | --- |
| kgp-F1 | ATCAGCGTGGTCGTGATACGATCGGACAGGGAGATTAC |
| kgp-R1 | TGCCGATCAACGTCTCAACTTTAAAACAATTTATGGTCGTGATTC |
| kgp-F2 | TGCTACGCCTGAATAAGTGAACTCGCTGTAAAGTAAATCTG |
| kpg-R2 | GATCTCAAGAAGATCCTTTGATAACGGAGGGAGGGAAAAG |
| rgpA-F1 | ATCAGCGTGGTCGTGAAACTTCTCTTGCAGCAGTAC |
| rgpA-R1 | TGCTACGCCTGAATAAGTGAACTCGCTATCAAGTAAATCTGTC |
| rgpA-F2 | TGCCGATCAACGTCTCAAGAGCAATCGAAACAAACTTG |
| rgpA-R2 | TCTCAAGAAGATCCTTTGATTCGTCTCGGATGGCATC |
| cat-F | TGAGACGTTGATCGGCAC |
| cat-R | TCACTTATTCAGGCGTAGCAAC |
| ori-F | TCACGACCACGCTGATG |
| ori-R | ATCAAAGGATCTTCTTGAGATCC |
| rgpB-F1 | ACGACGGCCAGTGAATTCGGAGCTCATGAGTTGGCTGTCATGAC |
| rgpB-R1 | TTGCTTGAATTAGTTTTTTATTTG |
| rgpB-F2 | AGCCTCTATGAATGGCAAAGTAATTCACACTGCAATTCTC |
| rgpB-R2 | TGACCATGATTACGCCAAAGCTTGGTTGAGGGTACGGTG |
| tetQ-F | ACAAATAAAAAACTAATTCAAGCAAATGAATATTATAAATTTAGGAATTCTTGCTC |
| tetQ-R | TGCCATTCATAGAGGCTATTATATG |
| kgp-F3 | ACGGCCAGTGAATTCGAGCTCTACGATCGGACAGGGAGATTAC |
| kgp-R3 | TGTTGCAAATACCGATGAGCAACTTTAAAACAATTTATGGTCGTGATTC |
| kgp-F4 | TGGCGATGGAGCGGAAACGTACTCGCTGTAAAGTAAATCTG |
| kgp-R4 | ACCATGATTACGCCAAAGCTTAACGGAGGGAGGGAAAAG |
| ermF-F | TGCTCATCGGTATTTGCAAC |
| ermF-R | ACGTTTCCGCTCCATC |
| F1 | ACATGGCTGTACGATTTCTAC |
| R1（F4） | AGGTACATTGAGCAACTGAC |
| F2（R3） | TGTGATGGCTTCCATGTCG |
| R2 | TCTCTTCATTGTCTCGGCTC |
| F3 | TGCTCCTGAGGTATGGAGAC |
| R4 | TGGCAGAAGATCAGGATGGAG |
| F5 | ACCTATAAGTATCCCAAGGCAG |
| R5 | ACAACGTCTCGAAGTCTC |
| F6 | ACAGACATTGCTGGAAGAAC |
| R6 | ACCACGTTCTACGTCGTAG |
| F7 | TGTCTATGAGGTAGGTCCTG |
| R7 | TCACAACCGACAACTTGAAC |

**Table S3 The primer sequences used for the analysis of gene expression in the colon.**

| **Primer target** | **Forward Sequence** | **Reverse sequence** |
| --- | --- | --- |
| SAA1 | CATTTGTTCACGAGGCTTTCC | GTTTTTCCAGTTAGCTTCCTTCATGT |
| SAA2 | TGATGCTGCCCAAAGG | GCCAGGAGGTCTGTAGTAA |
| SAA3 | GCAACTACTGGGTTGAGATA | ATTCAGCACATTGGGATG |
| RORγT | TGTCCTGGGCTACCCTACTG | GTGCAGGAGTAGGCCACATT |
| IL-6 | ACTTCCATCCAGTTGCCTTCTTGG | TTAAGCCTCCGACTTGTGAAGTGG |
| IL-17 | CAAGAAATCCTGGTCCTTCG | GAGCATCTTCTCCAACCTGAA |
| TGF-β | CTCCCGTGGCTTCTAGTGC | GCCTTAGTTTGGACAGGATCTG |
| Muc2 | CAACAAGCTTCACCACAATCTC | CAGACCAAAAGCAGCAAGGT |
| Occludin | TCACTTTTCCTGCGGTGACT | GGGAACGTGGCCGATATAATG |
| ZO-1 | AGGACACCAAAGCATGTGAG | GGCATTCCTGCTGGTTACA |
| GAPDH | AGGTCGGTGTGAACGGATTTG | TGTAGACCATGTAGTTGAGGTCA |
